# Supplementary material for: A modular cell-free protein biosensor platform using split T7 RNA polymerase
Source: Sci Adv. 2025 Feb 21;11(8):eado6280. doi: 10.1126/sciadv.ado6280 (PMC11844732; doi:10.1126/sciadv.ado6280)
Supplement: Supplementary file 1 — Supplementary Text Figs. S1 to S25 Tables S1 to S7 References [file sciadv.ado6280_sm.pdf]

Science Advances
AAAS

Supplementary Materials for

A modular cell-free protein biosensor platform using split T7 RNA polymerase

Megan A. McSweeney *et al.*

Corresponding author: Mark P. Styczynski, mark.styczynski@chbe.gatech.edu

Sci. Adv. **11**, eado6280 (2025)
DOI: 10.1126/sciadv.ado6280

This PDF file includes:

Supplementary Text
Figs. S1 to S25
Tables S1 to S7
References

Supplementary Text

During this study, and after most of the data were collected, we noticed mutations in the gene for T7RNAPC on several plasmids. For full transparency, we have included Table S1 that indicates which mutations were present on each plasmid used to generate the data in every figure. None of the plasmids used in this study have mutations in the NB, MB, or DARPin sequences. Additionally, there were no mutations in any plasmids encoding for the T7RNAPN or T7RNAPNev fragments. Plasmids not listed in this table did not have any mutations.

After identifying mutations in these plasmids, we corrected the mutations on three NB-T7RNAPC plasmids to test how these mutations impact TLISA functionality. Correcting the mutations on the LaG2-T7RNAPC plasmid resulted in a dysfunctional sensor (Fig. S6A). We decreased the reporter plasmid concentration to reduce leak but still did not observe any eGFP detection under any conditions (Fig. S6B). For the eGFP sensor that uses a MB fusion to the T7RNAPC (T7RNAPNev-LaG2/GS2-T7RNAPC), correcting the plasmid mutations improved the rate of reaction, and was still able to detect 500 nM eGFP (Fig. S6C). The eGFP sensor that uses a DARPin fusion to the T7RNAPC (T7RNAPNev-LaG2/3G86.32-T7RNAPC) also failed to detect eGFP after the mutations were corrected (Fig. S6D). In summary, while specific sensors can be more or less robust to these mutations, reversion of mutation V118M was most often deleterious and mutation G537R was generally less disruptive to performance.

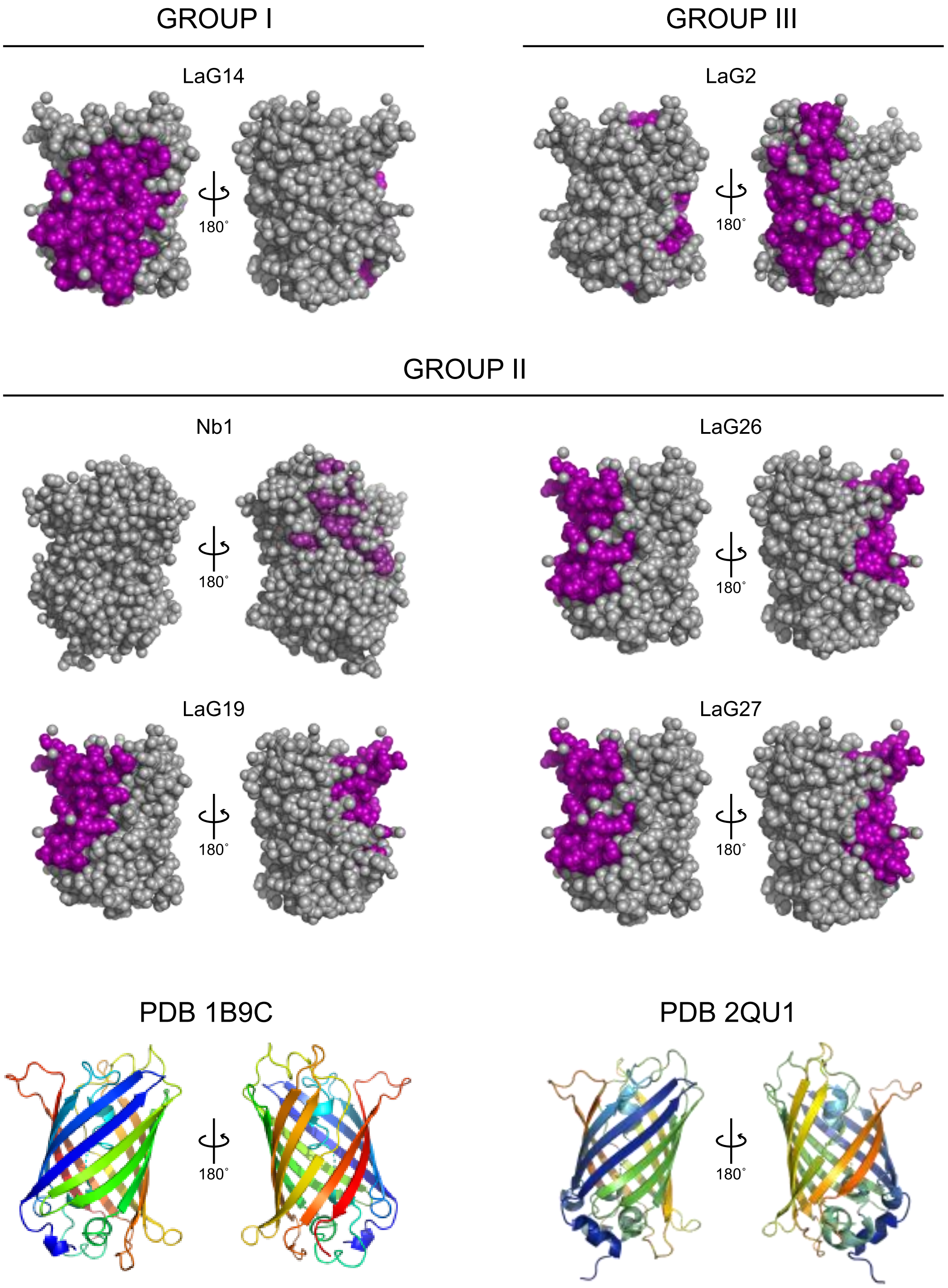

Fig. S1.

Visualization of the anti-eGFP NB epitopes. GFP molecule is shown in gray, and residues that interact with each NB are shown in purple. Binding residues for Nb1 were determined by Kubala et al. (33) and mapped onto PBD 2QU1. The remaining NBs were determined by Fridy *et al.* (25) and mapped onto PDB 1B9C. Illustrations were made using PyMol.

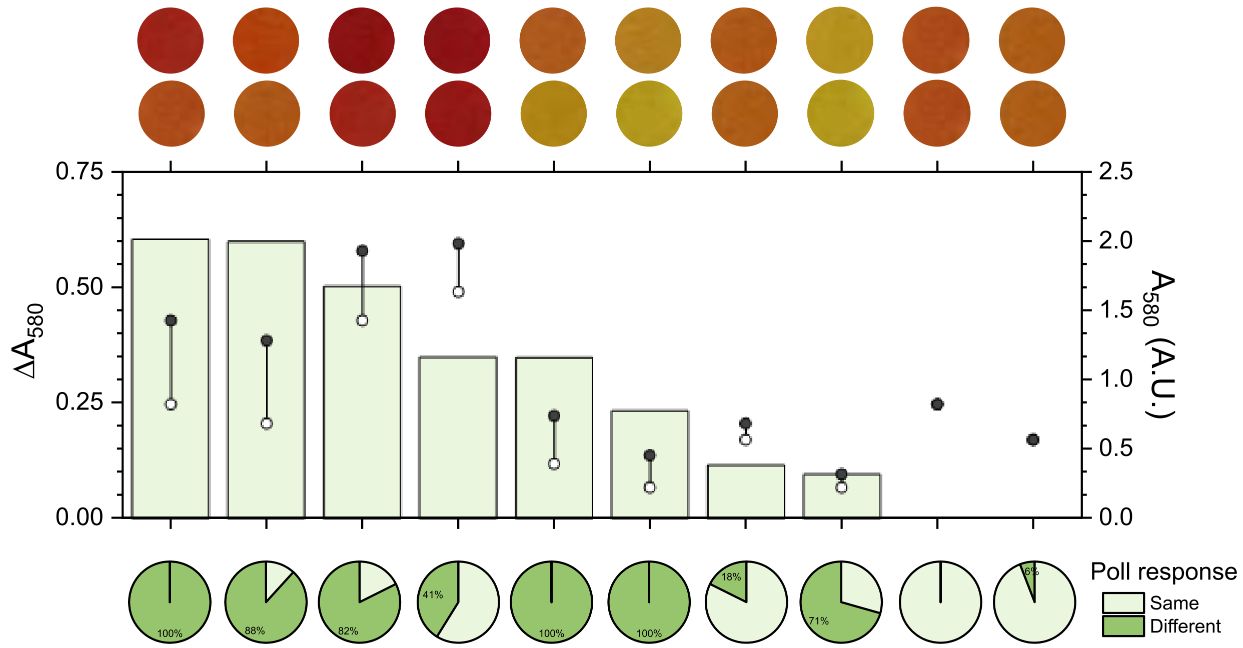

Fig. S2.

Determining visible limit of detection. 17 people were shown 10 different pairs of reaction photos (top) in randomized order and asked whether they thought the colors were the same or different. Plotted are the absorbance values at 580 nm (symbols, right y axis) and the difference in absorbance values (bars, left y axis) for each pair. Two of the pairs were the same exact color (right two pairs). The poll responses (bottom pie charts) indicate that pairs with greater differences in absorbance values are more universally distinguishable. With the exception of one pair of colors, most of the responses identified ΔA580 values greater than 0.233 as different colors (first data points). Two colors with lower A580 values (i.e., more yellow) are more visually distinguishable even with lower ΔA580 values.

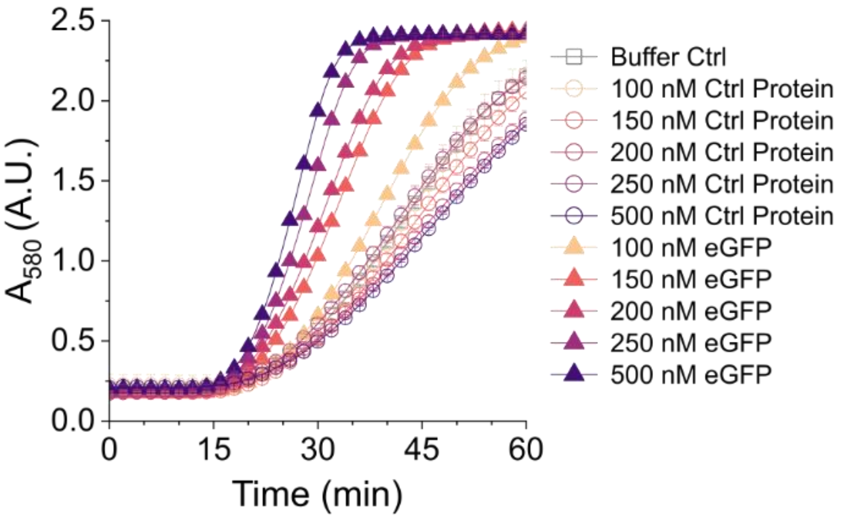

Fig. S3.

Detection range and LOD of the T7RNAP$_{Nev}$-LaG2/LaG14-T7RNAP$_{C}$ TLISA. Absorbance data with increasing concentrations of eGFP or control protein show an LOD of 100 nM eGFP after one hour. All reactions had 0.1 nM pT7LacZ. mCherry was used as the control protein. Bars represent the arithmetic mean ± standard deviation of n=3 technical replicates.

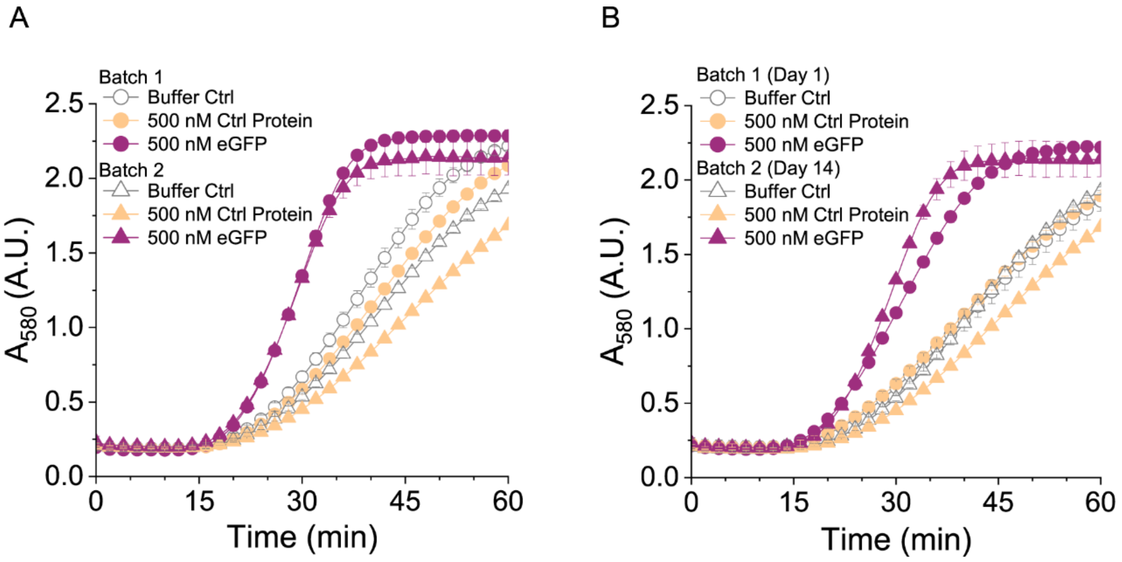

Fig. S4.

TLISA performance has minimal variation across different batches of crude lysate and different reaction days. (A) Absorbance data for a $T7RNAP_{Nev}$-NB1/LaG2-$T7RNAP_C$ TLISA using two different batches of in-house prepared crude lysate. (B) Absorbance data for a $T7RNAP_{Nev}$-NB1/LaG2-$T7RNAP_C$ TLISA using two different batches of in-house prepared crude lysate performed two weeks apart. Symbols represent the arithmetic mean ± standard deviation of n=3 technical replicates.

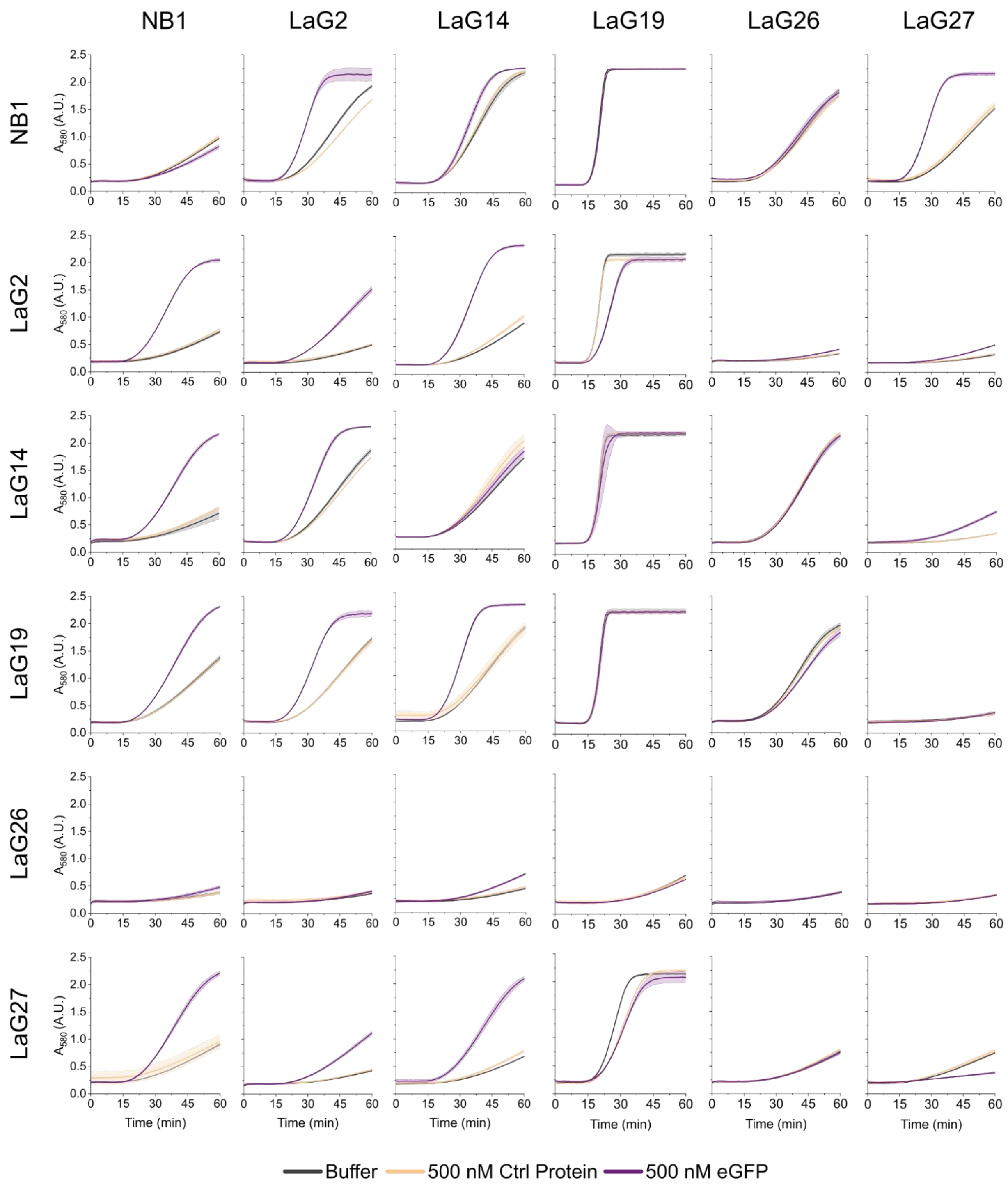

Fig. S5.

Absorbance data corresponding to the data in Figure 2A. Columns represent different $T7RNAP_C$ NB fusions and rows represent different $T7RNAP_{Nev}$ NB fusions. Lines and shaded areas represent the arithmetic mean ± standard deviation of n=3 technical replicates. Consistent with Figures 2B and 2C, the purple curve is for 500 nM eGFP, the yellow curve is for 500 nM control protein (mCherry), and the gray curve is for the buffer-only control.

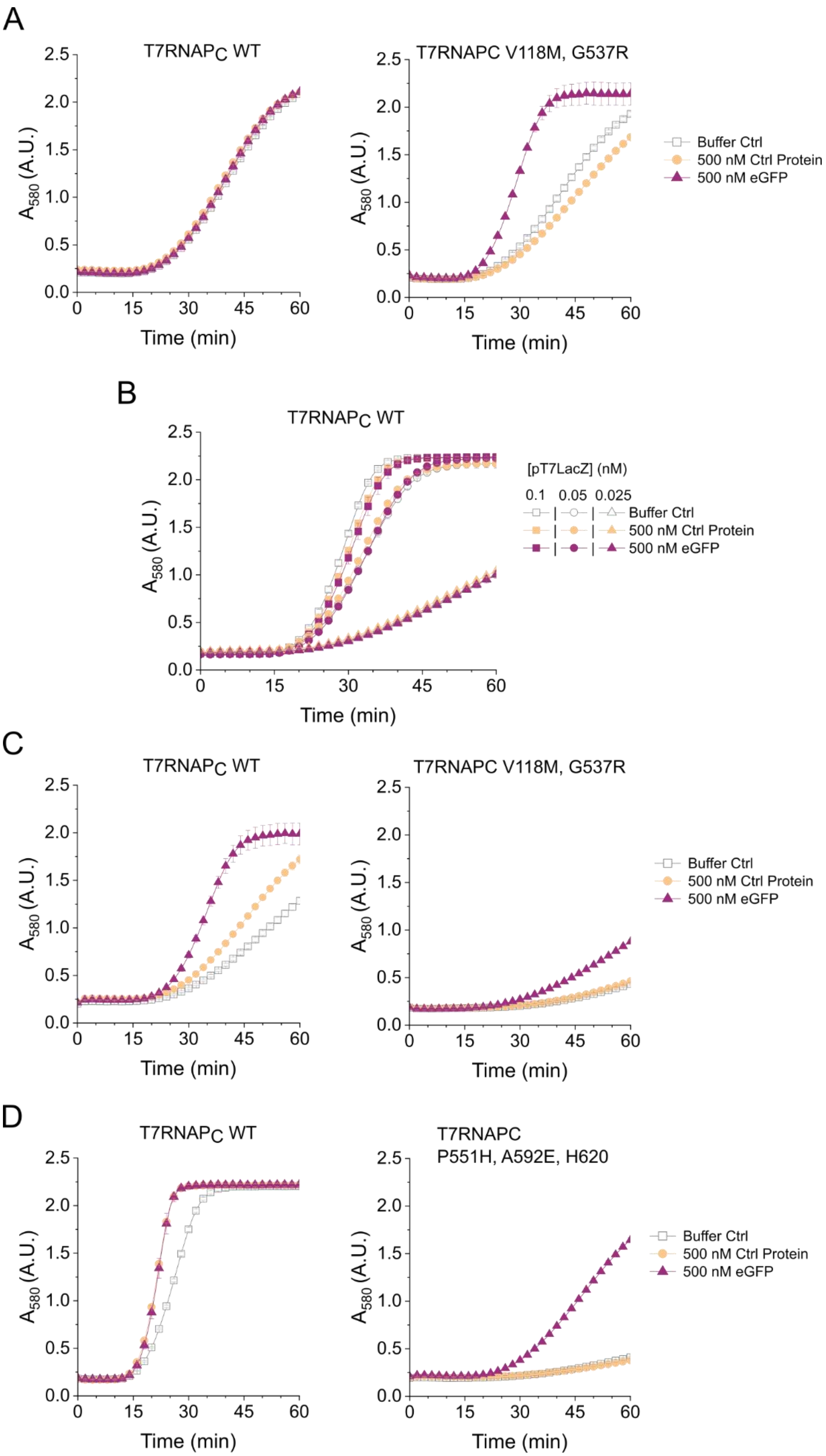
A
T7RNAPC WT
T7RNAPC V118M, G537R
A580 (A.U.)
Time (min)
Buffer Ctrl
500 nM Ctrl Protein
500 nM eGFP
B
T7RNAPC WT
[pT7LacZ] (nM)
0.1 0.05 0.025
Buffer Ctrl
500 nM Ctrl Protein
500 nM eGFP
C
T7RNAPC WT
T7RNAPC V118M, G537R
D
T7RNAPC WT
T7RNAPC
P551H, A592E, H620

Fig. S6.

TLISA performance when using either the WT T7RNAPC or the identified T7RNAPC mutations. (A) $T7RNAP_{Nev}$-NB1/LaG2-$T7RNAP_C$ eGFP TLISA with either (left) WT $T7RNAP_C$ or (right) mutated $T7RNAP_C$. (B) $T7RNAP_{Nev}$-NB1/LaG2-$T7RNAP_C$ eGFP TLISA using the WT $T7RNAP_C$ with a range of different pT7LacZ reporter plasmid concentrations. (C) $T7RNAP_{Nev}$-LaG2/GS2-$T7RNAP_C$ eGFP TLISA with either (left) WT $T7RNAP_C$ or (right) mutated $T7RNAP_C$. (D) $T7RNAP_{Nev}$-LaG2/3G86.32-$T7RNAP_C$ eGFP TLISA with either (left) WT $T7RNAP_C$ or (right) mutated $T7RNAP_C$.

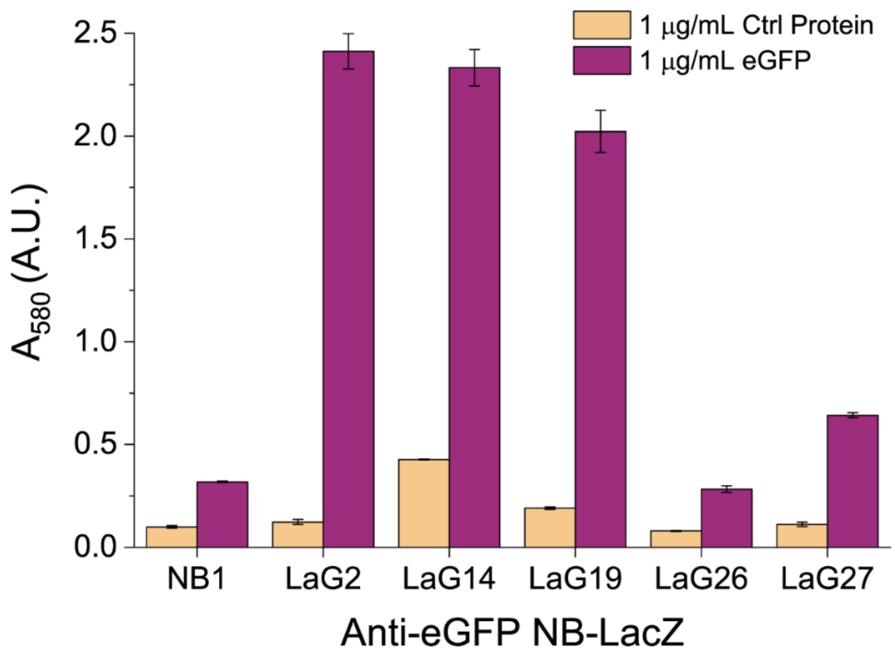

Fig. S7.

Direct ELISA validation of anti-eGFP NB expression and functionality in a CFE system. Anti-eGFP NBs were translationally fused to LacZ. TTR was used as the control protein. Bars and error bars represent the arithmetic mean ± standard deviation of n=3 technical replicates.

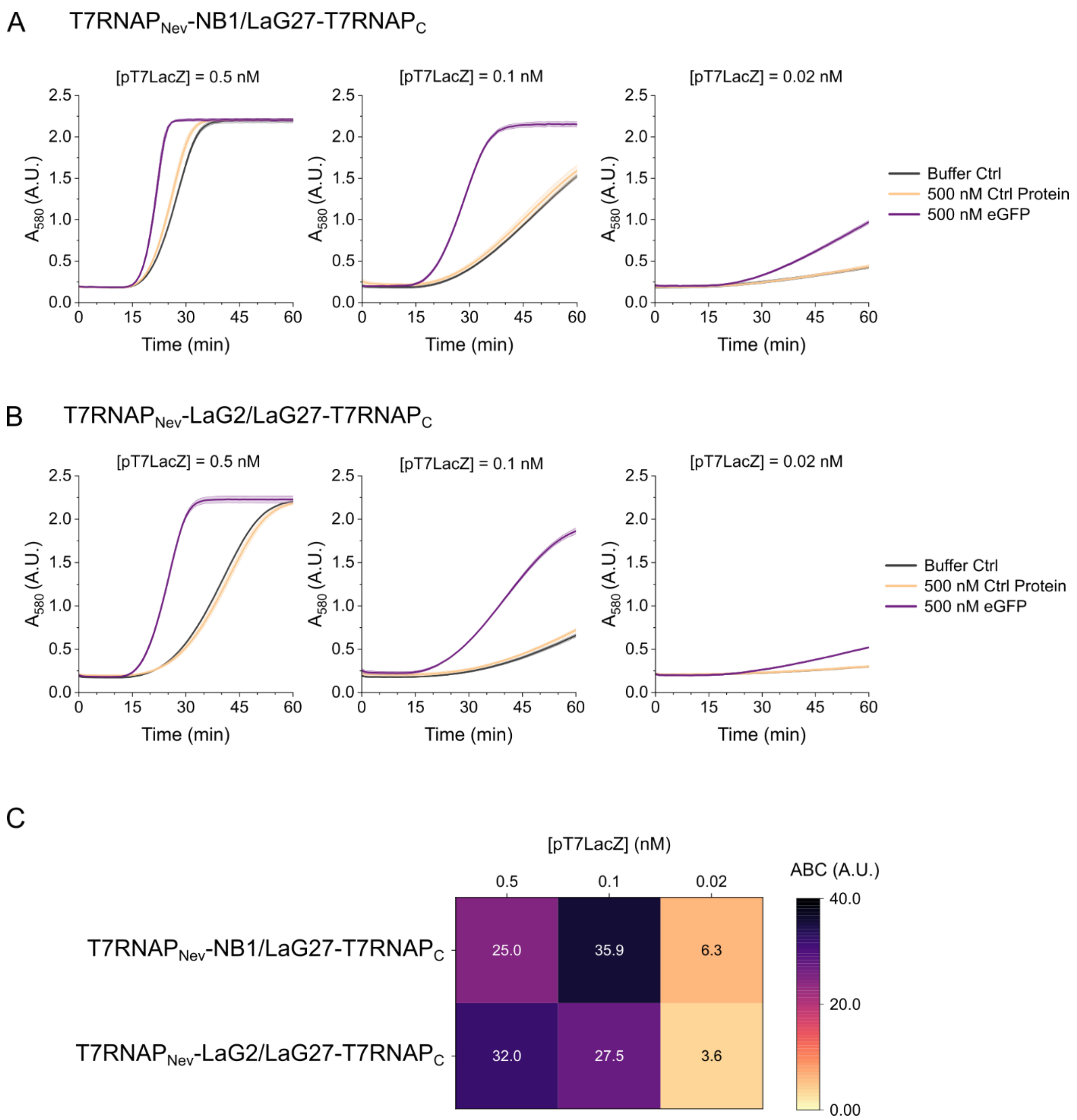

Fig. S8.

Optimal reporter plasmid concentration can vary across sensors. Absorbance data for (A) T7RNAP$_{Nev}$-NB1/LaG27-T7RNAP$_C$ and (B) T7RNAP$_{Nev}$-LaG2/LaG27-T7RNAP$_C$ with either 0.5 nM, 0.1 nM, or 0.02 nM pT7LacZ reporter plasmid. (C) Heatmap of ABC values for both sensors at all reporter plasmid concentrations. Shaded areas represent the standard deviation of the mean of n=3 technical replicates.

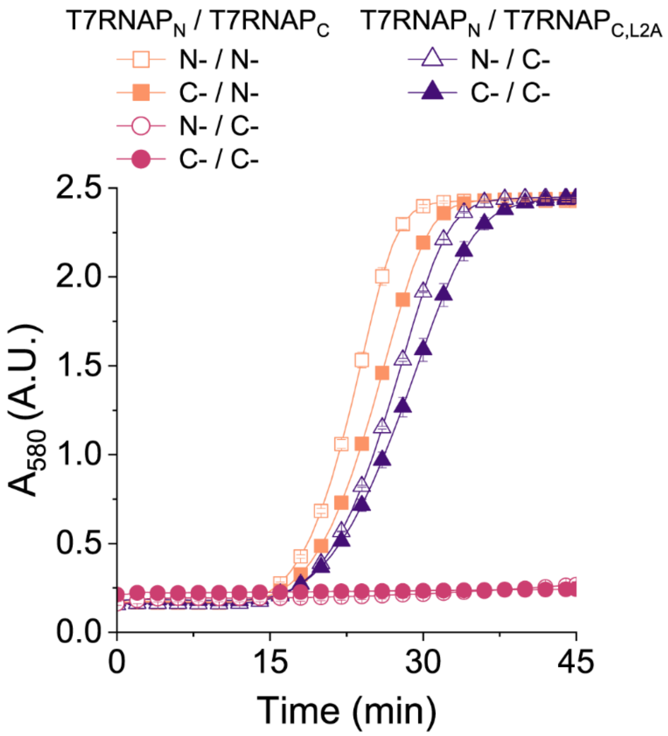

Fig. S9.

Spontaneous reassembly of T7RNAP fragments with NBs fused to different termini. All $T7RNAP_N$ fragments here are wild type to enable spontaneous reassembly. The evolved $T7RNAP_{C,L2A}$ variant enables C-terminal fusions. All reactions contained 0.1 nM pT7LacZ. Symbols represent the arithmetic mean ± standard deviation of n=3 technical replicates.

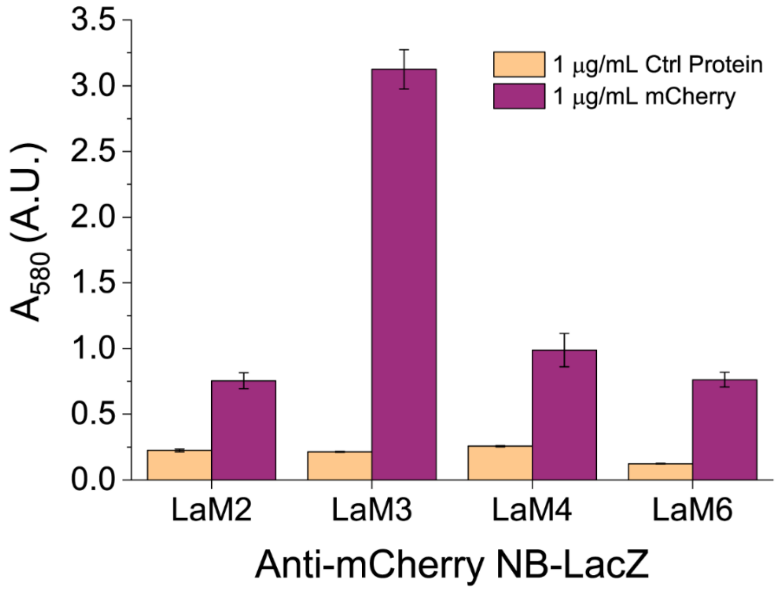

Fig. S10.

Direct ELISA validation of anti-mCherry NB expression in a CFE system. TTR was used as the control protein. Bars and error bars represent the arithmetic mean ± standard deviation of n=3 technical replicates.

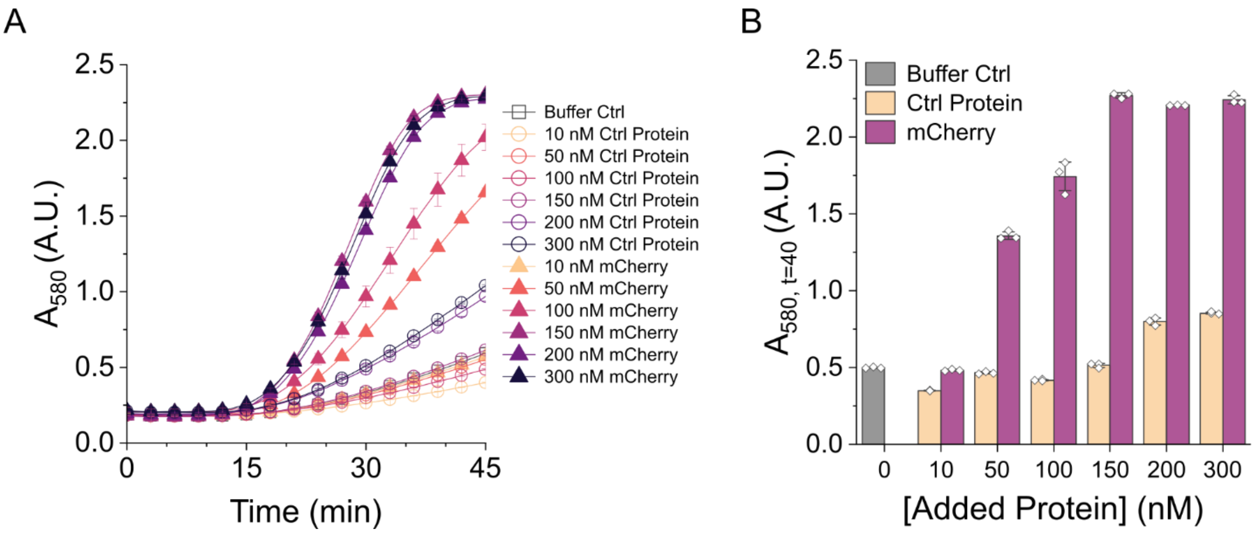

Fig. S11.

Detection range and LOD of the T7RNAP$_{\mathrm{Nev}}$-LaM4/LaM2-T7RNAP$_{\mathrm{C}}$ TLISA. (A) Absorbance data with increasing concentrations of mCherry or control protein showing an LOD of 50 nM mCherry. (B) Absorbance values at 40 minutes showing the detection range. All reactions had 0.05 nM pT7LacZ. TTR was used as the control protein. Bars represent the arithmetic mean ± standard deviation of n=3 technical replicates (white diamonds).

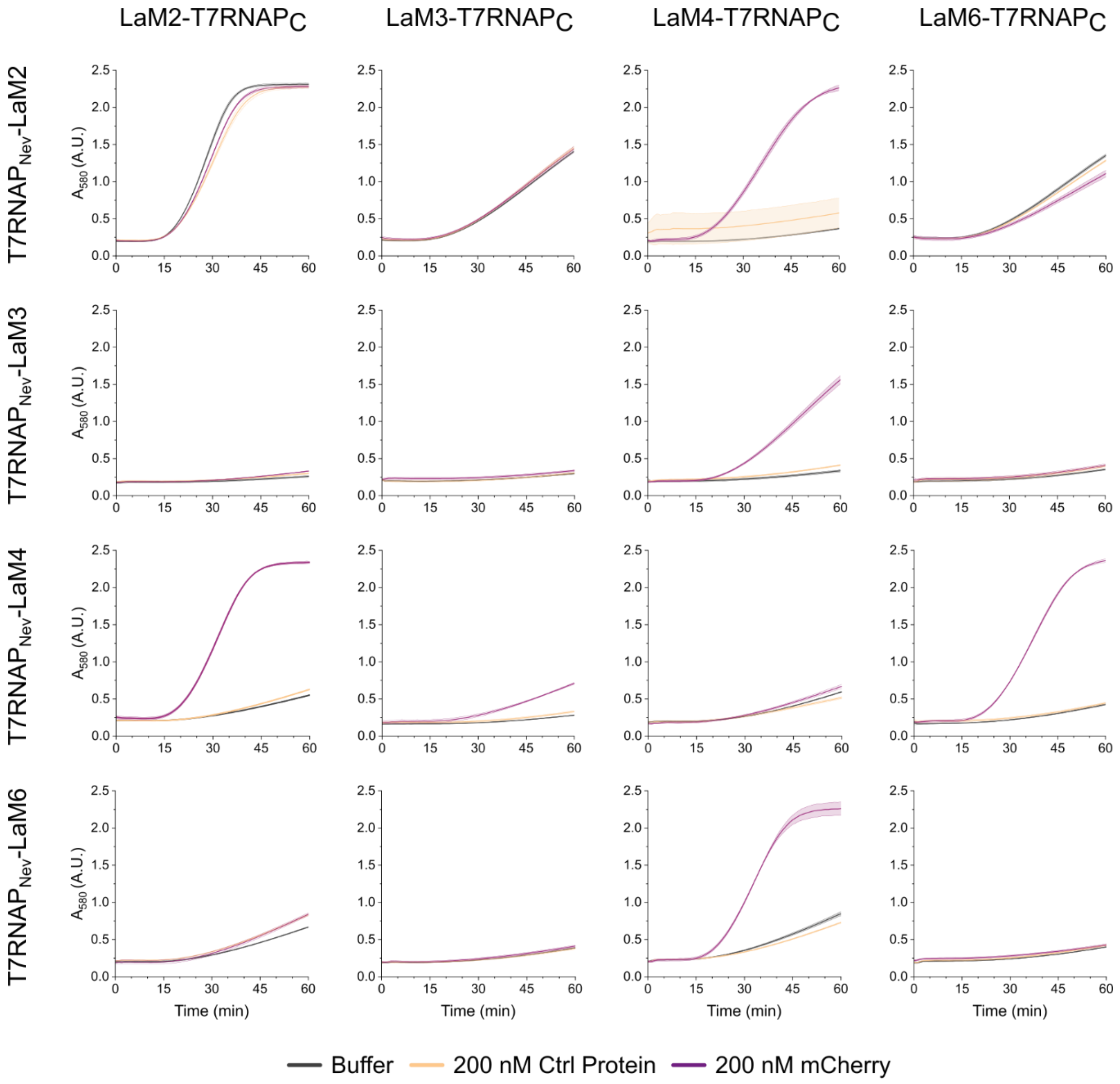

Fig. S12.

Absorbance data corresponding to the data in Figure 4B. Columns represent different T7RNAP$_C$ NB fusions and row represent different T7RNAP$_{Nev}$ NB fusions. Shaded areas represent the standard deviation of the mean of n=3 technical replicates. Consistent with Figure 4A, the purple curve is for 200 nM mCherry, the yellow curve is for 200 nM control protein (eGFP), and the gray curve is for the buffer-only control.

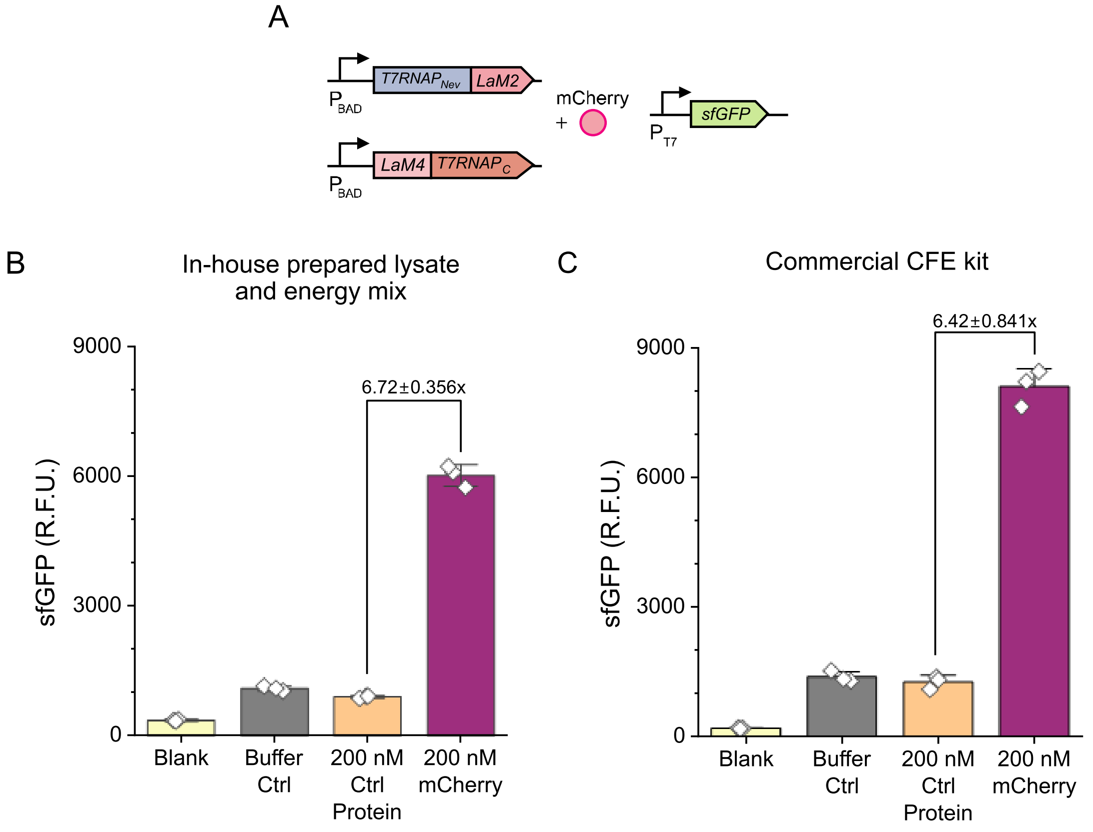

Fig. S13.

TLISA can use different protein outputs and different *in vitro* transcription and translation systems. (A) Sensor circuit for the T7RNAP$_{Nev}$-LaM2/LaM4-T7RNAP$_{C}$ mCherry TLISA using sfGFP as a protein reporter. Fluorescent measurements (ex. 485 nm, em. 510 nm) after 3 hours of incubation at 37 °C showing detection of mCherry when using (B) an in-house prepared crude lysate-based reaction and when using (C) the commercial myTXTL Sigma 70 kit. Blank represents the background fluorescence of the cell-free reaction and contains everything in the buffer control condition expect pT7sfGFP plasmid. 200 nM TTR was used as the control protein. Bars represent the arithmetic mean ± standard deviation of n=3 technical replicates (white diamonds).

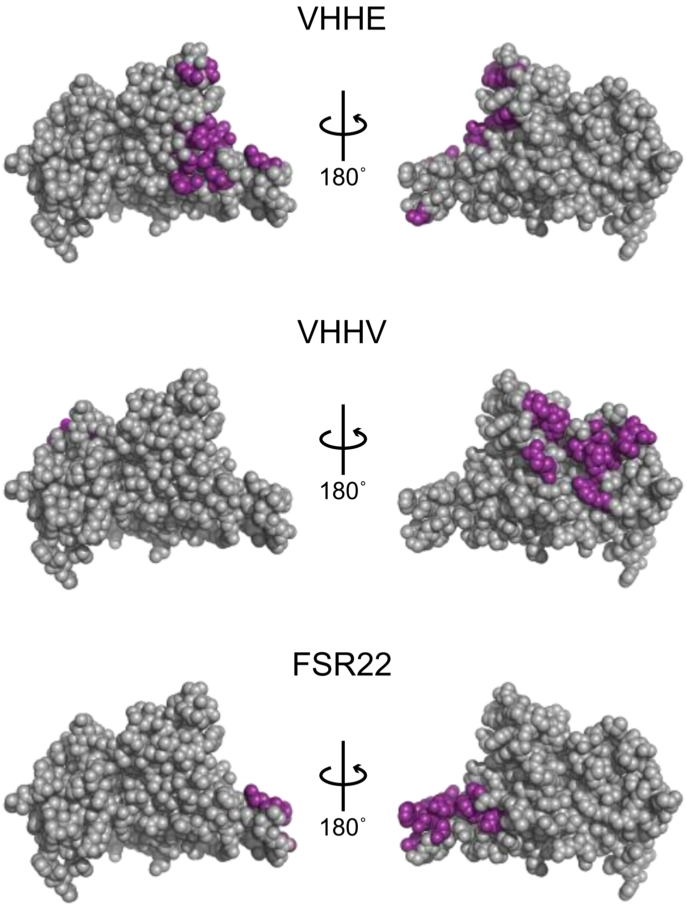

Fig. S14.

Visualization of the anti-RBD NB and DARPin binding sites. RBD molecule (PDB 6M0J) is shown in gray, and residues that interact with each NB or DARPin are shown in purple. Binding residues for VHHE and VHHV were determined by Koenig *et al*. (40) and the binding residues for FSR22 were determined by Chonira *et al*. (41). Illustrations were made using PyMol.

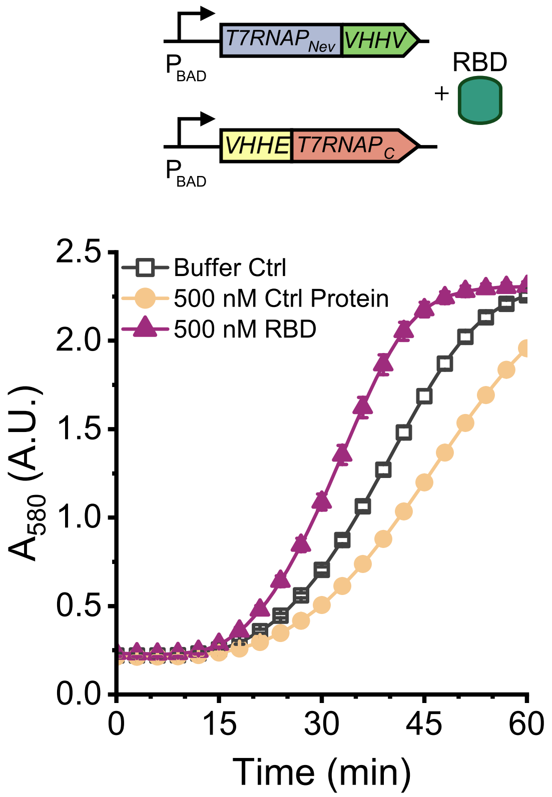

Fig. S15.

T7RNAPNev-VHHV/VHHE-T7RNAPC SARS-CoV-2 RBD TLISA. 500 nM mCherry was used as the control protein for all experiments. Symbols represent the arithmetic mean ± standard deviation of n=3 technical replicates.

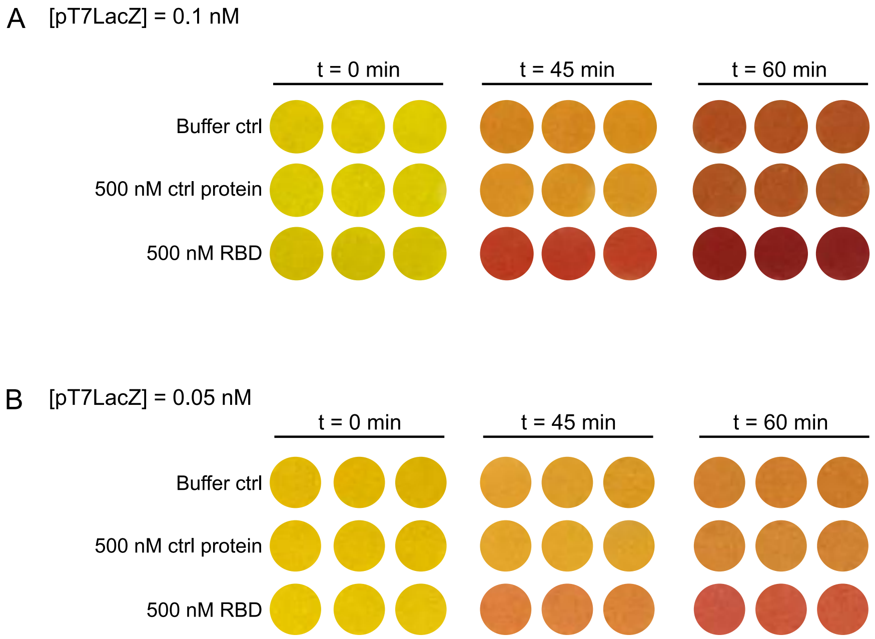

Fig. S16.

Visual detection of SARS-CoV-2 RBD. (A) Photos of visible reaction colors at different time points for the $T7RNAP_{Nev}$-FSR22/VHHV-$T7RNAP_{C}$ SARS-CoV-2 RBD TLISA using 0.1 nM pT7LacZ. Reactions with 500 nM RBD are more red than reactions containing an off-target control protein (mCherry) or just protein buffer after 45 minutes. Detection is still distinguishable at 60 minutes but leaky expression in the off state makes interpretation more difficult. (B) Using 50% lower pT7LacZ (0.05 nM) in this TLISA reaction reduces leak, making reactions appear more visually distinct for a longer period of time. However, doing so also decreases the overall reaction rate.

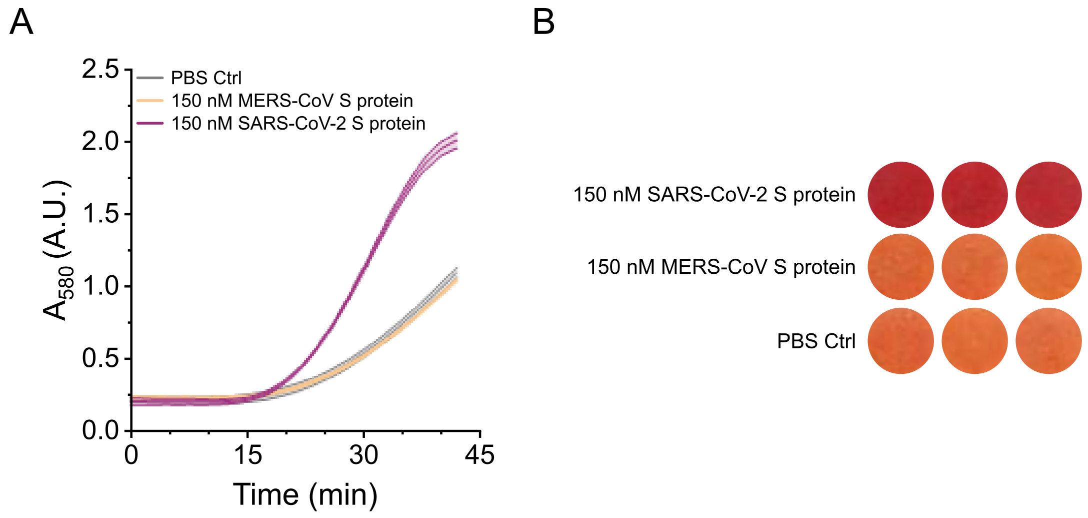

Fig. S17.

TLISA detection of the SARS-CoV-2 S protein. (A) Absorbance data of the $T7RNAP_{Nev}$-FSR22/VHHV-$T7RNAP_C$ showing detection of 150 nM SARS-CoV-2 S protein. (B) Pictures of visible reaction colors after 45 minutes of incubation. Despite background expression in the PBS and off-target protein (MERS CoV-2 S) controls, reactions containing 150 nM SARS-CoV-2 S protein are clearly more red.

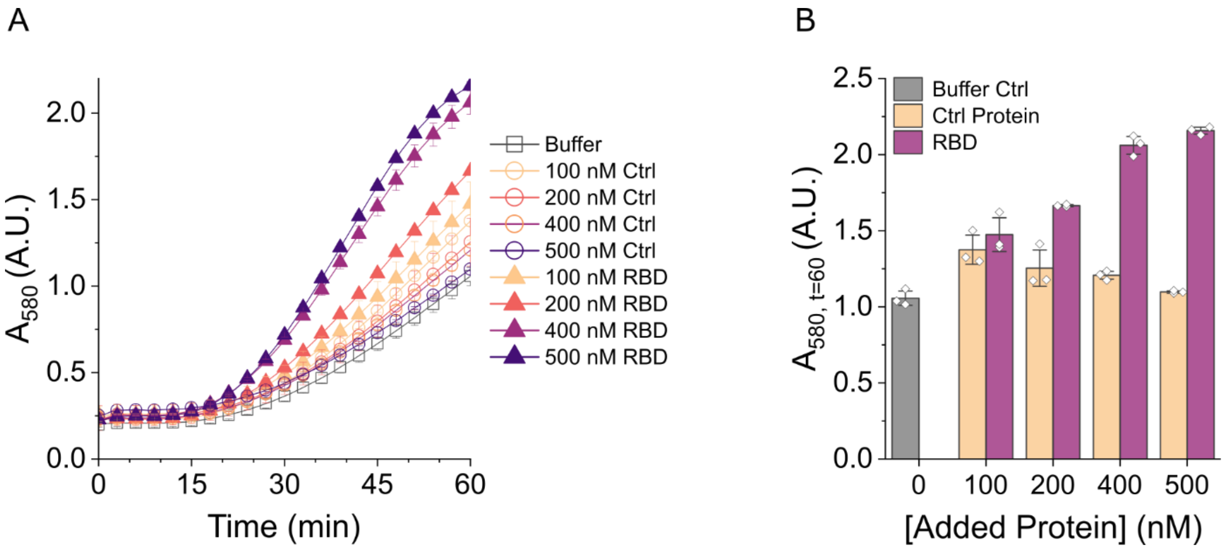

Fig. S18.

Determining the detection range and LOD of the T7RNAP$_{Nev}$-FSR22/VHHV-T7RNAP$_C$ biosensor for SARS-CoV-2 RBD. (A) Absorbance data with increasing concentrations of RBD or control protein showing an LOD of 200 nM RBD. (B) Absorbance values after 60 minutes of incubation at 37 °C. All reactions had 0.1 nM pT7LacZ. mCherry was used as the control protein. Bars represent the arithmetic mean ± standard deviation of n=3 technical replicates (white diamonds).

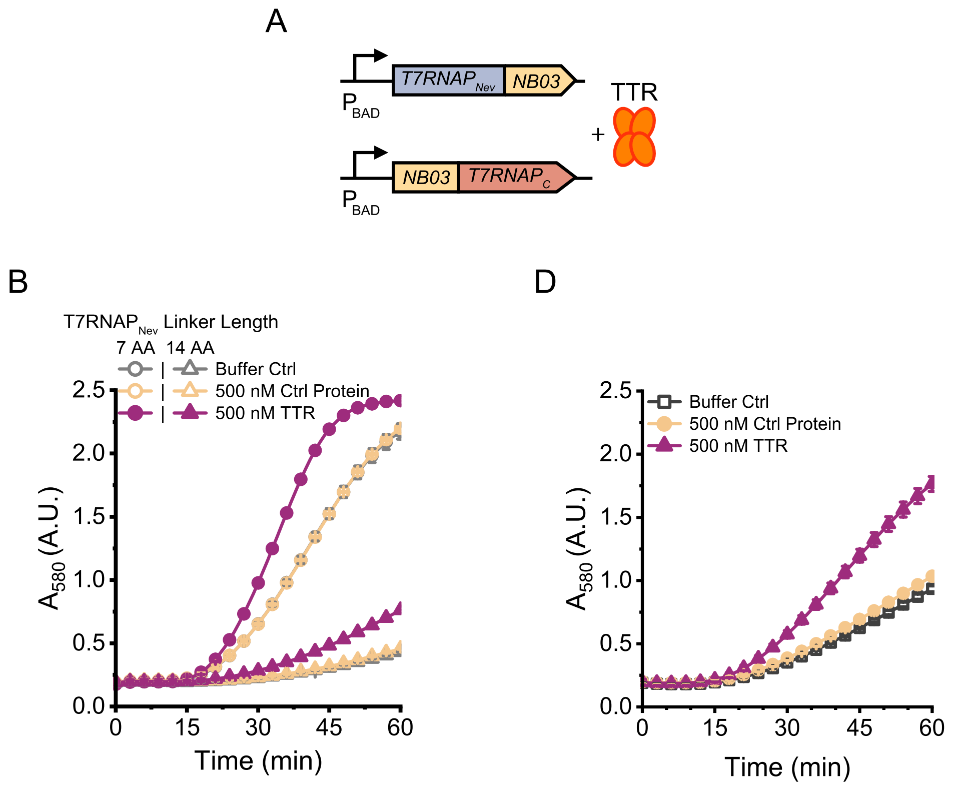

C

Linker Length (AA)	ABC (A.U.)
7	16.7577
14	6.7934

E

[pT7LacZ] (nM)	ABC (A.U.)
0.12	16.7577
0.05	15.8448

Fig. S19.

TTR sensor tuning. (A) Genetic sensing circuit for the TTR TLISA. (B) Absorbance data for TTR data with either a 7 AA linker (circles) or a 14 AA linker (triangles) on the T7RNAP$_{\mathrm{Nev}}$-NB03 fragment with 0.12 nM pT7LacZ. (C) ABC values for the data in (B). (D) Absorbance data for the TTR sensor using the 7 AA linker on the T7RNAP$_{\mathrm{Nev}}$-NB03 fragment with 0.05 nM pT7LacZ. (E) ABC values for 7 AA linker data in (B) and (D). 500 nM mCherry was used as the control protein for all experiments. Symbols represent the arithmetic mean ± standard deviation of n=3 technical replicates.

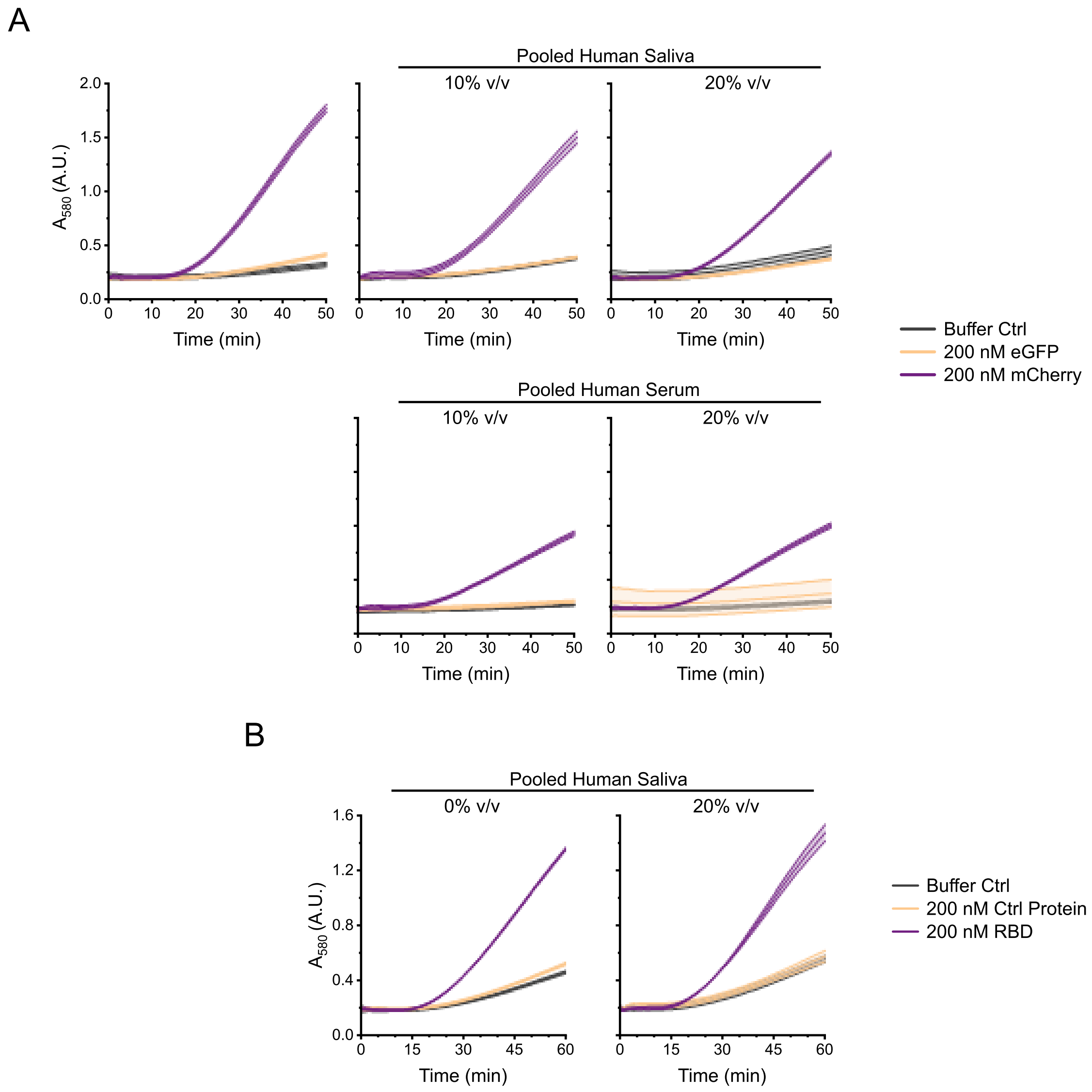

Fig. S20.

Absorbance time course data corresponding to Figures 6B and 6C. (A) Absorbance data for the T7RNAP$_{Nev}$-LaM4/LaM2-T7RNAP$_C$ mCherry TLISA in various concentrations of either pooled human saliva or serum as presented in Figure 6B. (B) Absorbance data for the T7RNAP$_{Nev}$-FSR22/VHHV-T7RNAP$_C$ SARS-CoV-2 RBD TLISA with and without pooled human saliva as presented in Figure 6C. Shaded areas represent the standard deviation of the mean of n=3 technical replicates.

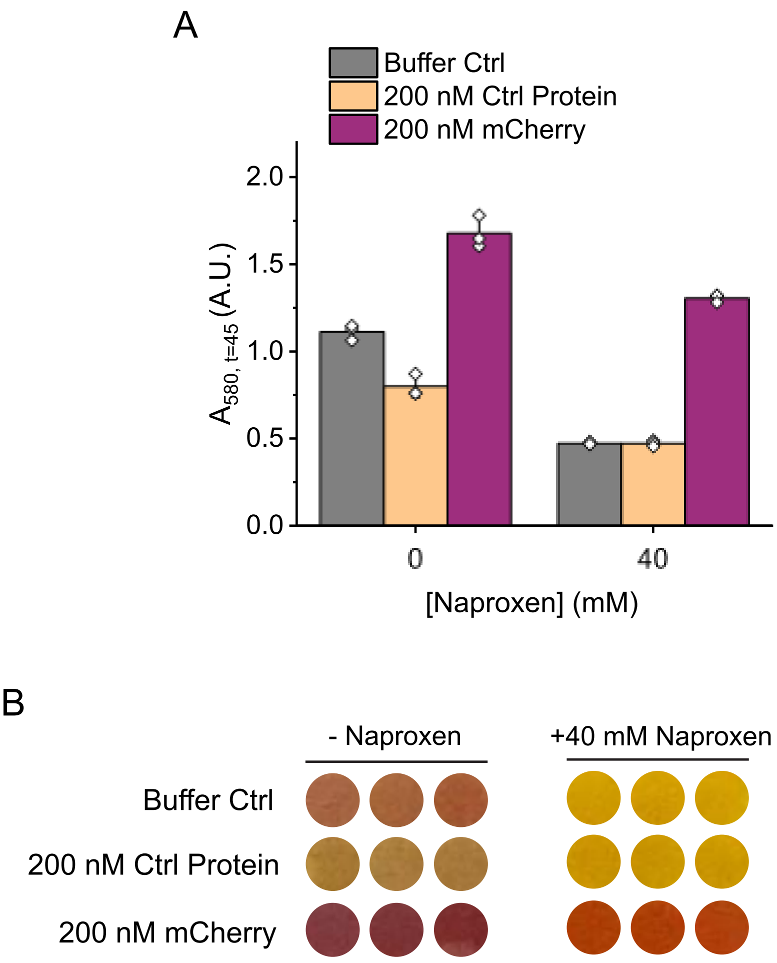

Fig. S21.

Naproxen restores vibrancy in colorimetric reactions in serum. (A) Absorbance values after 45 minutes of incubation showing detection of 200 nM mCherry in 20% pooled human serum with and without added naproxen. Naproxen is known to quench transcription, which explains why final absorbance values are lower when naproxen is added. (B) Pictures of reactions after 45 minutes of incubation showing that the addition of naproxen restores vibrancy back to reaction colors. Bars represent the arithmetic mean ± standard deviation of n=3 technical replicates (white diamonds).

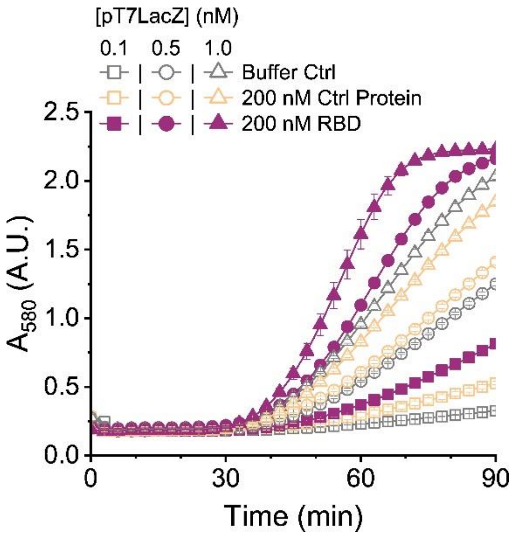

Fig. S22.

Increasing concentrations of pT7LacZ improves rate of reaction when sensing at room temperature. Absorbance data for T7RNAP$_{Nev}$-FSR22/VHHV-T7RNAP$_{C}$ SARS-CoV-2 RBD TLISA reaction in 20% v/v pooled human saliva incubated at 25 °C. 200 nM mCherry was used as the control protein. Symbols and error bars represent the arithmetic mean ± standard deviation of n=3 technical replicates.

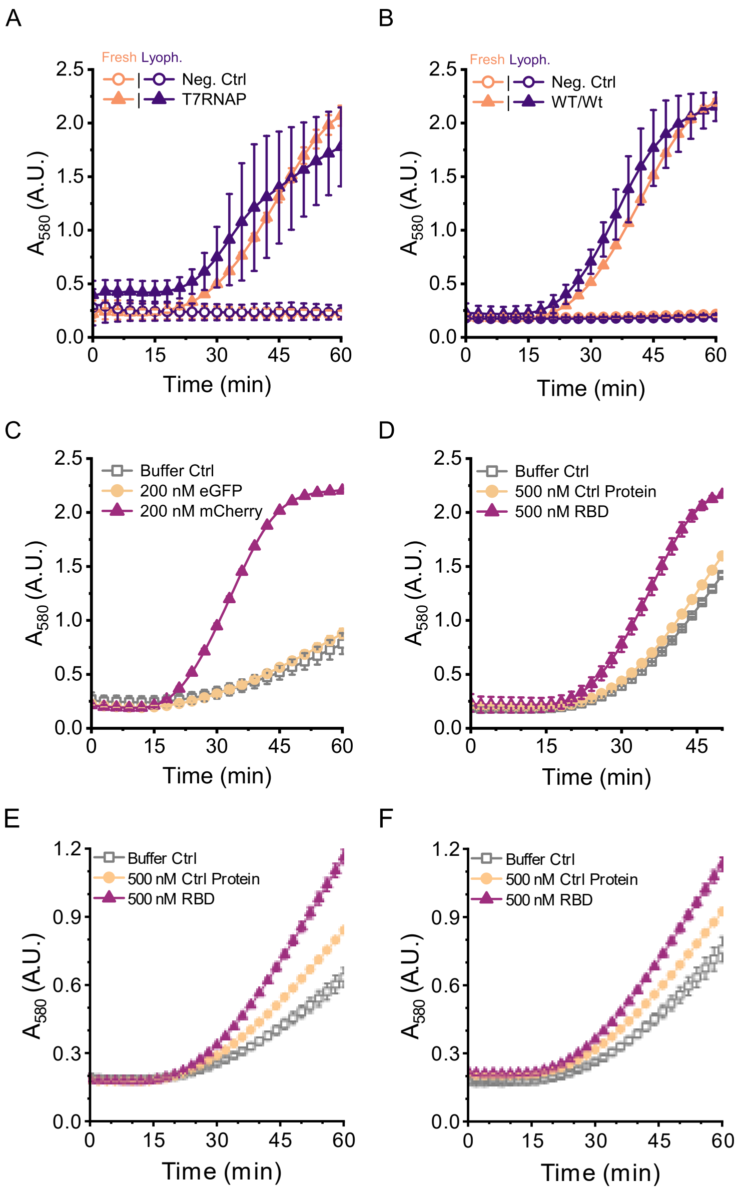

Fig. S23.

Successful lyophilization of cell-free reactions after 1 hour pre-expression reactions. (A) Lyophilized reactions after pre-expression of either a plasmid encoding full T7RNAP or an empty reaction (neg. ctrl) and rehydrated with 0.05 nM pT7LacZ. (B) Lyophilized reactions after pre-expression of plasmids encoding WT split T7RNAP fragments or an empty reaction (neg. ctrl) and rehydrated with 0.05 nM pT7LacZ. (C) Lyophilized T7RNAPNev-LaM4/LaM2-T7RNAPC mCherry TLISA rehydrated with 0.1 nM pT7LacZ. (D) Lyophilized T7RNAPNev-FSR22/VHHV-T7RNAPC SARS-CoV-2 RBD TLISA rehydrated with 0.5 nM pT7LacZ. (E) Lyophilized T7RNAPNev-FSR22/VHHV-T7RNAPC SARS-CoV-2 RBD TLISA rehydrated with 0.5% v/v RNAse Inhibitor and 0.75 nM pT7LacZ and 0% v/v human saliva or (F) 20% v/v human saliva, corresponding to Figure 6D. Symbols represent the arithmetic mean ± standard deviation of n=3 technical replicates.

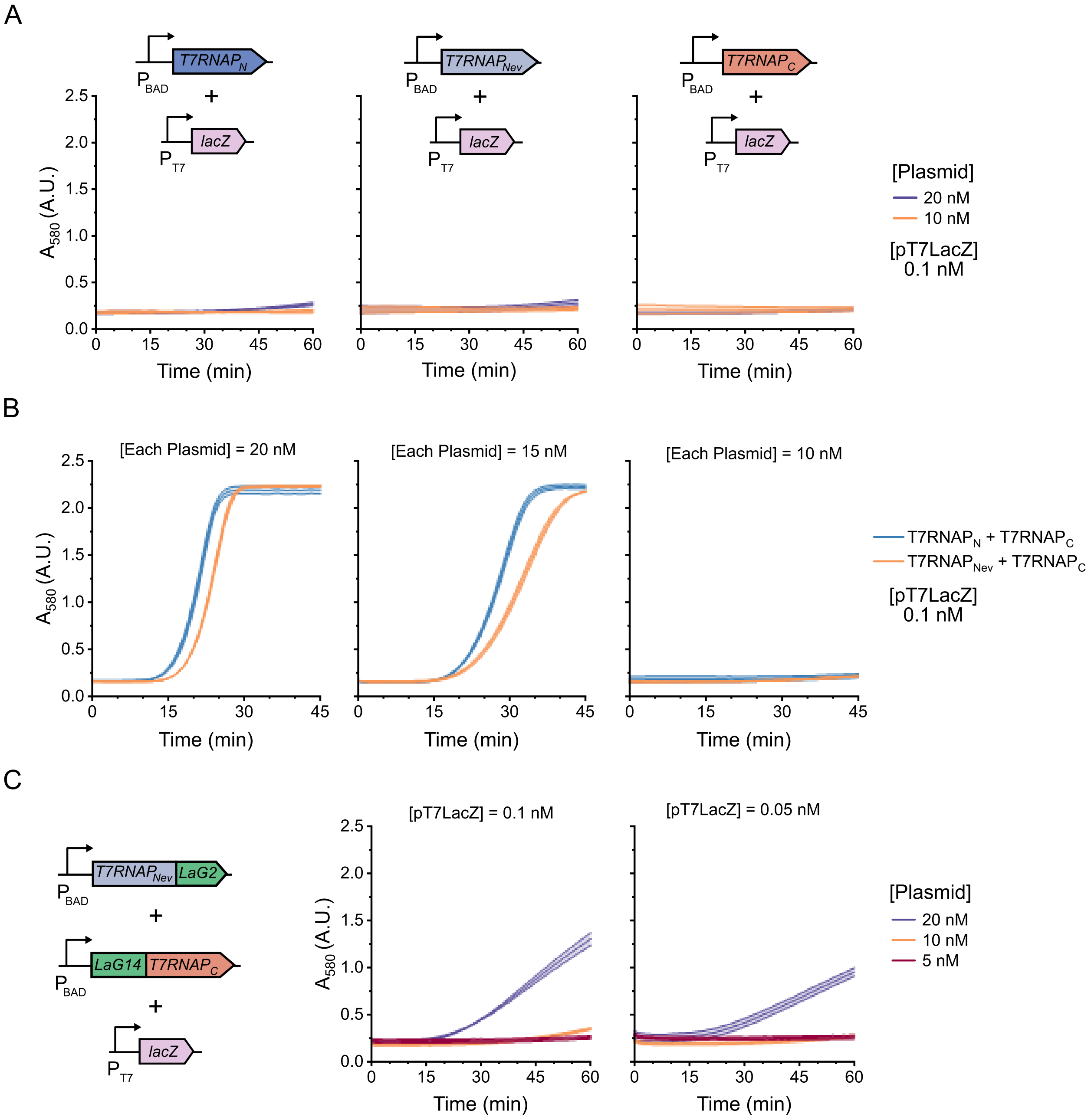

Fig. S24.

Leak assessment of the split T7RNAP in a CFE system. (A) The wt $T7RNAP_N$ fragment (left), the $T7RNAP_{Nev}$ fragment (middle), and the $T7RNAP_C$ fragment (right) alone have negligible activity at 20 nM and 10 nM of plasmid. (B) Relative to the wt $T7RNAP_N$ fragment, $T7RNAP_{Nev}$ has high levels of spontaneous reassembly with $T7RNAP_C$. (C) In the absence of antigen, the $T7RNAP_{Nev}$ and $T7RNAP_C$ fragments with different NB fusions have some spontaneous reassembly. Lower concentrations of T7RNAP fragments and pT7LacZ result in less LacZ activity. Shaded areas represent the standard deviation of the mean of n=3 technical replicates.

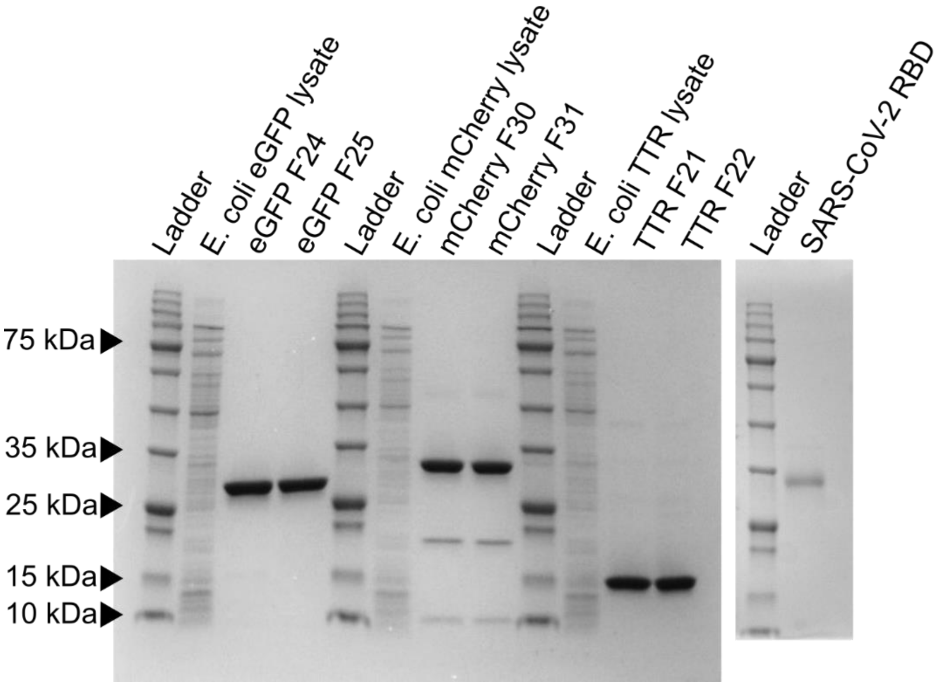

Fig. S25.

SDS-PAGE analysis of purified proteins used in this study. The additional bands and high apparent MW of mCherry (Lanes F30 and F31) are expected and not evidence of impurities; the bands appearing at 20 kDa and 10 kDa are a result of mCherry fragmentation due to denaturing by boiling (*37*, *60*)**.**

**Plasmid**	**Mutation(s)**	**Figure(s)**
NB1-T7RNAP$_C$	V118M, G537R	2A, S5
LaG2-T7RNAP$_C$	V118M, G537R	2A, 3A-D, S4-S6
LaG14-T7RNAP$_C$	V118M	1C, 1D, 2A, S3-S5, S24
LaG27-T7RNAP$_C$	V118M, G537R	2A, S5, S8
GS2-T7RNAP$_C$	V118M, G537R	S6
3G86.32-T7RNAP$_C$	P551H, A592E, H620N	2C, S6
NB1-Linker7-T7RNAP$_C$	V118M, G537R	3A
NB1-Linker28-T7RNAP$_C$	V118M, G537R	3A
LaM2-T7RNAP$_C$	V118M	4A, 4B, S11, S12, S23
LaM3-T7RNAP$_C$	V118M	4B, S12
LaM4-T7RNAP$_C$	V118M	4B, S12, S13, S20, S21
LaM6-T7RNAP$_C$	V118M	4B, S12
VHHE-T7RNAP$_C$	V118M, G537R	S15
NB03-T7RNAP$_C$	V118M	5C, S19

Table S1.
Summary of plasmids containing mutations used in this study.

	**ELISA**	**LFA**	**TLISA**
**LOD**	0.01 pM - 0.320 nM (*61*)	0.9 pM - 0.3 nM (42)	50 nM - 500 nM
**Cost per test**	$6.59 - $7.12 (*62*)	$5 - $45 (*63*)	$0.22 (*64*)
**Reaction time**	5 hrs - >24 hrs	15 mins - 20 mins (*63*)	35 mins - 60 mins
**Ease of development**	Low	Low	High
**User-friendly?**	No	Yes	Yes
**Labor intensive?**	Yes	No	No
**Shelf stable?**	No	Yes	Yes
**Reporter options**	Color change<br>Fluorescence	Color change	Color change<br>Fluorescence
**Quantitative readout?**	Yes	No	Semi-quantitative

Table S2.

Comparing conventional protein detection assays with TLISA. LODs can depend on many factors and can vary greatly for a single type of assay. Costs provided do not include any labor costs. The cost for TLISA was determined using previously calculated reagent costs for lysate-based cell-free reactions and accounts for one test reaction and one control reaction. Ease of development takes into consideration the time required to create antigen-binding domains and implement into the assay. ELISAs and LFAs both use monoclonal antibodies, which have a long development timeline and thus add to the complexity of developing ELISAs and LFAs for new targets. TLISA uses non-antibody affinity domains, which are simpler to generate and engineer. User-friendliness considers the need for laboratory equipment and long incubation steps to run the assay.

Name	Type	Target antigen	Amino acid sequence	Ref.
NB1	Nanobody	eGFP	QVQLVESGGALVQPGGSLRLSCAASGFPVNRYSMRWYRQAPGKEREWVAGMSSAGDRSSYEDSVKGRFTISRDDARNTVYLQMNSLKPEDTAVYYCNVNVGFEYWGQGTQVTVSSK	(*33*)
LaG2	Nanobody	eGFP	AQVQLVESGGGLVQAGGSLRLSCAASGRTFSNYAMGWFRQAPGKEREFVAAISWTGVSTYYADSVKGRFTISRDNDKNTVYVQMNSLIPEDTAIYYCAAVRARSFSDTYSRVNEYDYWGQGTQVTV	(*25*)
LaG14	Nanobody	eGFP	AQVQLVESGGGLVQAGGSLRLSCAASGRTYSISAMGWFRQAPGKEREFVAGISRSGGTTYYADPVKGRFTISRDNAKNTVYLQMNSLKPEDTAVYYCAARARGWTTFPAREIEYDYWGQGTQVTV	(*25*)
LaG19	Nanobody	eGFP	AQVQLVESGGGLVQAGGSLRLSCAASGPTGAMAWFRQAPGKEREFVGGISRSGTDTYYVDSVKGRFTIDRDNAKNTVYLQMNSLKPEDTAVYYCAARRSQILFTSRTDYEFWGQGTQVTV	(*25*)
LaG26	Nanobody	eGFP	AQVQLVESGGGLVQAGASMRLSCAASGITFSLYHWVWFRQAAGREHEFVAGIIRSGGETLSADSVKDRFIISRDDAKNTLYLQMNMLQPEDTATYYCAATHRADWYSSAFREYIFRGQGTQVTVS	(*25*)
LaG27	Nanobody	eGFP	ADVQLVESGGGLVQAGGSLRLSCTASGLTISTYNIGWFRQAPGKEREFVGIIIRNGDTTYYADSVKGRFTISRDNAKNTVYLQMNSVKPADAAVYSCGATVRAGAAAEQYNSYIFRGQGTQVTV	(*25*)
GS2	Monobody	eGFP	VSSVPTKLEVVAATPTSLLISWDAPAVTVDHYYITYGETGHYWYYQAFAVPGSKSTATISGLSPGVDYTITVYAPFSVPVMSPISINYRT	(*34*)
3G86.32	DARPin	eGFP	RGSGSDLGKKLLEAARAGQDDEVRILMANGADVNALDRFGLTPLHLAAQRGHLEIVEVLLKCGADVNAADLWGQTPLHLAATAGHLEIVEVLLKYGADVNALDLIGKTPLHLTAIDGHLEIVEVLLKHGADVNAQDKFGKTAFDISIDNGNEDLAEILQKLN	(*35*)
LaM2	Nanobody	mCherry	AQVQLVESGGGLVQAGGSLRLSCATSGFTFSDYAMGWFRQAPGKEREFVAAISWSGHVTDYADSVKGRFTISRDNVKNTVYLQMNSLKPEDTAVYSCAAAKSGTWWYQRSENDFGSWGQGTQVTVSKEAI	(*25*)

LaM3	Nanobody	mCherry	AQVQLVQSGGGLVQAGGSLRLSCAASGRTFSDIAVGWFRQTPGKEREFVAAISWSGLIINYGDSVEDRFTISRDNAKSAVYLQMNSLKPEDTAVYYCAARIGMNYYYAREIEYPYWGQGTQVTVSKCY	(*25*)
LaM4	Nanobody	mCherry	AQVQLVESGGSLVQPGGSLRLSCAASGRFAESSSMGWFRQAPGKEREFVAAISWSGGATNYADSAKGRFTLSRDNTKNTVYLQMNSLKPDDTAVYYCAANLGNYISSNQRLYGYWGQGTQVTVSSPFT	(*25*)
LaM6	Nanobody	mCherry	AQVQLVESGGGLVQAGGSLRLSCVASGSAPSFFAMAWYRQSPGNERELVAALSSLGSTNYADSVKGRFTISMDNAKNTVYLQMNNVNAEDTAVYYCAAGDFHSCYARKSCDYWGQGTQVTVS	(*25*)
VHHE	Nanobody	SARS CoV-2 RBD	QVQLVETGGGFVQPGGSLRLSCAASGVTLDYYAIGWFRQAPGKEREGVSCIGSSDGRTYYSDSVKGRFTISRDNAKNTVYLQMNSLKPEDTAVYYCALTVGTYYSGNYHYTCSDDMDYWGKGTQVTVSSGSGLNDIFEAQKIEWHE	(*40*)
VHHV	Nanobody	SARS CoV-2 RBD	QVQLVETGGGLVQPGGSLRLSCAASGFTFSSYAMGWARQVPGKGLEWVSYIYSDGSTEYQDSVKGRFTISRDNAKSTVYLQMNSLKPEDTAVYYCATEGSLGGWGRDFGSWGQGTQVTVSS	(*40*)
FSR22	DARPin	SARS CoV-2 RBD	GSSSSGMEQKLISEEDLDGYIPEAPRDGQAYVRKDGEWVLLSTFLGGGGSLQGGGGSLQGSDLGKKLLEAARAGQDDEVRILMANGADVNACDPSGITPLHLAADKGHLEIVEVLLKYGADVNAMDVWGRTPLHLAAFTGHLEIVEVLLKYGADVNACDLNGYTPLHLAAGRGHLEIVEVLLKNGAGVNAQDKFGKTAFDISIDNGNEDLAEILQSSS	(*41*)
NB03	Nanobody	TTR	QVQLQESGGGSVQAGGSLRLSCAASGNTYSYKVIGWFRQAPGKEREGIAAIYTGGVSSTRYADSVKGRFTISRDNTKNAVYLQMNSLKPEDTAMYYCAAGPLYDSTWFRAEKYNYWGQGTQVTVSS	(*47*)

Table S3.
Sequences of nanobodies, monobodies, and DARPins used in this study.

**Name**	**Sequence**	**Ref.**
T7RNAP$_{N}$	NTINIAKNDFSDIELAAIPFNTLADHYGERLAREQLALEHESYEMG<br>EARFRKMFERQLKAGEVADNAAAKPLITTLLPKMIARINDWFEEVK<br>AKRGKRPTAFQFLQEIKPEAVAYITIKTTLACLTSADNTTVQAVAS<br>AIGRAIEDEARFGRIRDLEAKHFKKNVEEQLNKRVGHVYK	(*31*)
T7RNAP$_{Nev}$	NTINIAKNDFSDIELAAIPLNTLADHYGERSARGQLALEHESYEMG<br>EARFRKMFECQLKAGKVADNAAAKPLITTLLPKMIARINDWFEEVK<br>AKRGRRPTAFKFLKEIKPEAVAYITIKTSLACLTSADNTTVQAVAS<br>AIGRTIEDEARFGRIRDLEAKHFKKNVEEQLNKRVGHVYK	(*32*)
T7RNAP$_{C}$	KAFMQVVEADMLSKGLLGGEAWSSWHKEDSIHVGVRCIEMLIESTG<br>MVSLHRQNAGVVGQDSETIELAPEYAEAIATRAGALAGISPMFQPC<br>VVPPKPWTGITGGGYWANGRRPLALVRTHSKKALMRYEDVYMPEVY<br>KAINIAQNTAWKINKKVLAVANVITKWKHCPVEDIPAIEREELPMK<br>PEDIDMNPEALTAWKRAAAAVYRKDKARKSRRISLEFMLEQANKFA<br>NHKAIWFPYNMDWRGRVYAVSMFNPQGNDMTKGLLTLAKGKPIGKE<br>GYYWLKIHGANCAGVDKVPFPERIKFIEENHENIMACAKSPLENTW<br>WAEQDSPFCFLAFCFEYAGVQHHGLSYNCSLPLAFDGSCSGIQHFS<br>AMLRDEVGGRAVNLLPSETVQDIYGIVAKKVNEILQADAINGTDNE<br>VVTVTDENTGEISEKVKLGTKALAGQWLAYGVTRSVTKRSVMTLAY<br>GSKEFGFRQQVLEDTIQPAIDSGKGLMFTQPNQAAGYMAKLIWESV<br>SVTVVAAVEAMNWLKSAAKLLAAEVKDKKTGEILRKRCAVHWVTPD<br>GFPVWQEYKKPIQTRLNLMFLGQFRLQPTINTNKDSEIDAHKQESG<br>IAPNFVHSQDGSHLRKTVVWAHEKYGIESFALIHDSFGTIPADAAN<br>LFKAVRETMVDTYESCDVLADFYDQFADQLHESQLDKMPALPAKGN<br>LNLRDILESDFAFA	(*31*)
T7RNAP$_{C,L2A}$	KAFMQAVEADMLSKGLLGGEAWSSWHKEDSIHVGVRCIEMLIESTG<br>MVSLHRQNAGVVGQDSETIELAPEYAEAIATRAGALAGISPMFQPC<br>VVPPKPWTGITGGGYWANGRRPLALVRTHSKKALMRYEDVYMPEVY<br>KAINIAQNTAWKINKKVLAVANVITKWKNCPVEDIPAIEREELPMK<br>PEDIDTNPEALTAWKRAAAAVYRKDKARKSRRISLEFMLEQANKFA<br>NHKAIWFPYNMDWRGRVYAVPMFNPQGNDMTKGLLTLAKGKPIGKE<br>GYYWLKIHGANCAGVDKVPFPERIKFIEENHENIMACAKSPLGNTW<br>WAEQDSPFCFLAFCFEYAGVQHHGLSYNCSLPLAYDESCSGIQHFS<br>AMLRDEVGGRAVNLIPSETVQDIYGIVAKKVNEILQADAINGTDNE<br>VVTVTDENTGEISEKVKLGTKALAGQWLAYGVTRSVTKRSVMTLAY<br>GSKEFGFRQQVLEDTIQPAIDSGKGLMFTQPNHAAGYMAKLIWESA<br>SVTVVAAVEAMNWLKSAAKLLAAEVKDKKTGEILRKRCSVHWVTPD<br>GFPVWQEYKKPIQTRLNLMFLGQFRLQPTINTNKDSEIDAHKQESG<br>IAPNFVHSQDGSHLRKTVVWAHEKYGIESFALIHDSFGTIPADAAN<br>LFKAVRETMVDTYESCDVLADFYDQFADQLHESQLDKMPALPAKGN<br>LNLRDILESDFAFA	(*36*)

Table S4.

Sequences of the T7RNAP fragments used in this study.

Name	Sequence
Linker14	GGSGSSGGSGSGSS
Linker7	GGSGSSG
Linker28	GGSGSSGGSGSGSSGGSGSSGGSGSGSS

Table S5.

Sequences of the flexible amino acid linkers used in this study. Linker14 was taken from literature as a functional linker sequence for fusing affinity domains to the split T7 RNAP (*31*). Linker7 is the first 7 AAs of Linker14. Linker28 is Linker14 doubled.

Name	Sequence
eGFP	6xHis Tag-eGFP<br><br>MHHHHHHASKGEELFTGVVPILVELDGDVNGHKFSVSGEGEGDATYGKLT<br>LKFICTTGKLPVPWPTLVTTLCYGVQCFSRYPDHMKRHDFFKSAMPEGYV<br>QERTIFFKDDGNYKTRAEVKFEGDTLVNRIELKGIDFKEDGNILGHKLEY<br>NYNSHNVYIMADKQKNGIKVNFKTRHNIEDGSVQLADHYQQNTPIGDGPV<br>LLPDNHYLSTQSALSKDPNEKRDHMVLLEFVTAAGITHGMDELYN
mCherry	6xHis Tag-mCherry<br><br>MHHHHHHVSKGEEDNMAIIKEFMRFKVHMEGSVNGHEFEIEGEGEGRPYE<br>GTQTAKLKVTKGGPLPFAWDILSPQFMYGSKAYVKHPADIPDYLKLSFPE<br>GFKWERVMNFEDGGVVTVTQDSSLQDGEFIYKVKLRGTNFPSDGPVMQKK<br>TMGWEASSERMYPEDGALKGEIKQRLKLKDGGHYDAEVKTTYKAKKPVQL<br>PGAYNVNIKLDITSHNEDYTIVEQYERAEGRHSTGGMDELYK
SARS-CoV2 RBD	Leader Peptide-RBD-Linker-SpyTag-6xHis Tag<br><br>METDTLLLWVLLLWVPGSTGDRVQPTESIVRFPNITNLCPFGEVFNATRF<br>ASVYAWNRKRISNCVADYSVLYNSASFSTFKCYGVSPTKLNDLCFTNVYA<br>DSFVIRGDEVRQIAPGQTGKIADYNYKLPDDFTGCVIAWNSNNLDSKVGG<br>NYNYLYRLFRKSNLKPFERDISTEIYQAGSTPCNGVEGFNCYFPLQSYGF<br>QPTNGVGYQPYRVVVLSFELLHAPATVCGPKKSTNLVKNKCVNFGGSGGS<br>AHIVMVDAYKPTKHHHHHH
Transthyretin (TTR)	6xHis Tag-TTR Monomer<br><br>MHHHHHHGPTGTGESKCPLMVKVLDAVRGSPAINVAVHVFRKAADDTWEP<br>FASGKTSESGELHGLTTEEQFVEGIYKVEIDTKSYWKALGISPFHEHAEV<br>VFTANDSGPRRYTIAALLSPYSYSTTAVVTNPKE
SARS-CoV-2 Hexapro S ectodomain (*65*)	S protein-foldon trimerization motif-6xHis Tag<br><br>MFVFLVLLPLVSSQCVNLTTRTQLPPAYTNSFTRGVYYPDKVFRSSVLHS<br>TQDLFLPFFSNVTWFHAIHVSGTNGTKRFDNPVLPFNDGVYFASTEKSNI<br>IRGWIFGTTLDSKTQSLLIVNNATNVVIKVCEFQFCNDPFLGVYYHKNNK<br>SWMESEFRVYSSANNCTFEYVSQPFLMDLEGKQGNFKNLREFVFKNIDGY<br>FKIYSKHTPINLVRDLPQGFSALEPLVDLPIGINITRFQTLLALHRSYLT<br>PGDSSSGWTAGAAAYYVGYLQPRTFLLKYNENGTITDAVDCALDPLSETK<br>CTLKSFTVEKGIYQTSNFRVQPTESIVRFPNITNLCPFGEVFNATRFASV<br>YAWNRKRISNCVADYSVLYNSASFSTFKCYGVSPTKLNDLCFTNVYADSF<br>VIRGDEVRQIAPGQTGKIADYNYKLPDDFTGCVIAWNSNNLDSKVGGNYN<br>YLYRLFRKSNLKPFERDISTEIYQAGSTPCNGVEGFNCYFPLQSYGFQPT<br>NGVGYQPYRVVVLSFELLHAPATVCGPKKSTNLVKNKCVNFNFNGLTGTG<br>VLTESNKKFLPFQQFGRDIADTTDAVRDPQTLEILDITPCSFGGVSVITP<br>GTNTSNQVAVLYQDVNCTEVPVAIHADQLTPTWRVYSTGSNVFQTRAGCL<br>IGAEHVNNSYECDIPIGAGICASYQTQTNSPGSASSVASQSIIAYTMSLG<br>AENSVAYSNNSIAIPTNFTISVTTEILPVSMTKTSVDCTMYICGDSTECS<br>NLLLQYGSFCTQLNRALTGIAVEQDKNTQEVFAQVKQIYKTPPIKDFGGF

	NFSQILPDPSKPSKRSPIEDLLFNKVTLADAGFIKQYGDCLGDIAARDLI<br>CAQKFNGLTVLPPLLTDEMIAQYTSALLAGTITSGWTFGAGPALQIPFPM<br>QMAYRFNGIGVTQNVLYENQKLIANQFNSAIGKIQDSLSSTPSALGKLQD<br>VVNQNAQALNTLVKQLSSNFGAISSVLNDILSRLDPPEAEVQIDRLITGR<br>LQSLQTYVTQQLIRAAEIRASANLAATKMSECVLGQSKRVDFCGKGYHLM<br>SFPQSAPHGVVFLHVTYVPAQEKNFTTAPAICHDGKAHFPREGVFVSNGT<br>HWFVTQRNFYEPQIITTDNTFVSGNCDVVIGIVNNTVYDPLQPELDSFKE<br>ELDKYFKNHTSPDVDLGDISGINASVVNIQKEIDRLNEVAKNLNESLIDL<br>QELGKYEQGSGYIPEAPRDGQAYVRKDGEWVLLSTFLGGLNDIFEAQKIE<br>WHEHHHHHH
MERS-CoV 2P S ectodomain (*66*)	S protein-foldon trimerization domain-6xHis Tag<br><br>MIHSVFLLMFLLTPTESYVDVGPDSVKSACIEVDIQQTFFDKTWPRPIDV<br>SKADGIIYPQGRTYSNITITYQGLFPYQGDHGDMYVYSAGHATGTTPQKL<br>FVANYSQDVKQFANGFVVRIGAAANSTGTVIISPSTSATIRKIYPAFMLG<br>SSVGNFSDGKMGRFFNHTLVLLPDGCGTLLRAFYCILEPRSGNHCPAGNS<br>YTSFATYHTPATDCSDGNYNRNASLNSFKEYFNLRNCTFMYTYNITEDEI<br>LEWFGITQTAQGVHLFSSRYVDLYGGNMFQFATLPVYDTIKYYSIIPHSI<br>RSIQSDRKAWAAFYVYKLQPLTFLLDFSVDGYIRRAIDCGFNDLSQLHCS<br>YESFDVESGVYSVSSFEAKPSGSVVEQAEGVECDFSPLLSGTPPQVYNFK<br>RLVFTNCNYNLTKLLSLFSVNDFTCSQISPAAIASNCYSSLILDYFSYPL<br>SMKSDLSVSSAGPISQFNYKQSFSNPTCLILATVPHNLTTITKPLKYSYI<br>NKCSRFLSDDRTEVPQLVNANQYSPCVSIVPSTVWEDGDYYRKQLSPLEG<br>GGWLVASGSTVAMTEQLQMGFGITVQYGTDTNSVCPKLEFANDTKIASQL<br>GNCVEYSLYGVSGRGVFQNCTAVGVRQQRFVYDAYQNLVGYYSDDGNYYC<br>LRACVSVPVSVIYDKETKTHATLFGSVACEHISSTMSQYSRSTRSMLKRR<br>DSTYGPLQTPVGCVLGLVNSSLFVEDCKLPLGQSLCALPDTPSTLTPASV<br>GSVPGEMRLASIAFNHPIQVDQLNSSYFKLSIPTNFSFGVTQEYIQTTIQ<br>KVTVDCKQYVCNGFQKCEQLLREYGQFCSKINQALHGANLRQDDSVRNLF<br>ASVKSSQSSPIIPGFGGDFNLTLLEPVSISTGSRSARSAIEDLLFDKVTI<br>ADPGYMQGYDDCMQQGPASARDLICAQYVAGYKVLPPLMDVNMEAAYTSS<br>LLGSIAGVGWTAGLSSFAAIPFAQSIFYRLNGVGITQQVLSENQKLIANK<br>FNQALGAMQTGFTTTNEAFHKVQDAVNNNAQALSKLASELSNTFGAISAS<br>IGDIIQRLDPPEQDAQIDRLINGRLTTLNAFVAQQLVRSESAALSAQLAK<br>DKVNECVKAQSKRSGFCGQGTHIVSFVVNAPNGLYFMHVGYYPSNHIEVV<br>SAYGLCDAANPTNCIAPVNGYFIKTNNTRIVDEWSYTGSSFYAPEPITSL<br>NTKYVAPQVTYQNISTNLPPPLLGNSTGIDFQDELDEFFKNVSTSIPNFG<br>SLTQINTTLLDLTYEMLSLQQVVKALNESYIDLKELGNYTYGSGYIPEAP<br>RDGQAYVRKDGEWVLLSTFLGGLNDIFEAQKIEWHEHHHHHH

Table S6.

Sequences of the protein antigens used in this study.

Name	Sequence (5' → 3')	Assembly Method
UniversalLaG-Cwt insert.F	atacccgtttttttgggctagcCCACAACGGTTTCCCTCTAGAAATA	Gibson Assembly
LaG14/LaG19/LaG27-Cwt insert.R	cccgctacctccgctactcccactgccaccGACGGTTACTTGTGTACCTTGC	Gibson Assembly
LaG26-Cwt insert.R	cccgctacctccgctactcccactgccaccACTTACCGTGACTTGGGTGC	Gibson Assembly
UniversalLaG-Cwt backbone.F	GGTGGCAGTGGGAGTAGC	Gibson Assembly
UniversalLaG-Cwt backbone.R	ctagagggaaaccgttgtggGCTAGCCCAAAAAAACGGGTATG	Gibson Assembly
Nev-LaG2 insert.F	cggaggtagcgggagtggcagcagtGCGCAGGTGCAGCTG	Gibson Assembly
Nev-LaG2 insert.R	gttagcagccggtcgacttaTACTGTAACTTGTGTACCCTGGCC	Gibson Assembly
Nev-LaG2 backbone.F	GTACACAAGTTACAGTAtaaGTCGACCGGCTGCTAACAAA	Gibson Assembly
Nev-LaG2 backbone.R	ACTCTACCAGCTGCACCTGCGCactgctgccactcccgc	Gibson Assembly
Nev-LaG14 insert.F	cggaggtagcgggagtggcagcagtGCTCAAGTGCAGCTTGTCGAAAG	Gibson Assembly
Nev-LaG14 insert.F	gctttgttagcagccggtcgacttaGACGGTTACTTGTGTACCTTGCC	Gibson Assembly
Nev-LaG14 backbone.F	ggcaaggtacacaagtaaccgtcTAAGTCGACCGGCTGCTAACAAAG	Gibson Assembly
Nev-LaG14 backbone.R	gcACTGCTGCCACTCCCG	Gibson Assembly
Nev-LaG19 insert.F	cggaggtagcgggagtggcagcagtGCGCAAGTGCAATTGGTTGAATC	Gibson Assembly
Nev-LaG26 insert.F	aggtagcgggagtggcagcagtGCACAGGTACAGCTTGTAGAGTCT	Gibson Assembly
Nev-LaG26 insert.R	ttgttagcagccggtcgacttaACTTACCGTGACTTGGGTGC	Gibson Assembly
Nev-LaG26 backbone.F	aggcacccaagtcacggtaagtTAAGTCGACCGGCTGCTAACAAA	Gibson Assembly
Nev-LaG26 backbone.R	cagactctacaagctgtacctgtgcACTGCTGCCACTCCCG	Gibson Assembly
Nev-LaG27 insert.F	ggaggtagcgggagtggcagcagtGCAGATGTTCAGCTTGTCGAATC	Gibson Assembly
3G86.32-Cwt insert.F	tttaagaaggagatatacatATGAGAGGATCGGGATCCGA	Gibson Assembly
3G86.32-Cwt insert.R	cccgctacctccgctactcCCACTGCCACCATTAAGCTTTTG	Gibson Assembly
3G86.32-Cwt backbone.F	GAAATCCTGCAAAAGCTTAATgGCTACTCCCACTGCCAC	Gibson Assembly

3G86.32-Cwt backbone.R	GGATCCCGATCCTCTCATATGTATATCTCC TTCTTAAAGTTAAACAAAA	Gibson Assembly
Nev-LaMuniversal insert.F	cggaggtagcgggagtggcagcagtGCGCA AGTACAACTGGTAGAGTC	Gibson Assembly
Nev-LaM2 insert.R	gctttgttagcagccggtcgacttaGATAG CCTCCTTTGATACCGTTACCT	Gibson Assembly
Nev-LaM3 insert.R	gctttgttagcagccggtcgacttaGTAAC ATTTGCTAACCGTCACTTGAGTG	Gibson Assembly
Nev-LaM4 insert.R	gctttgttagcagccggtcgacttaAGTGA AAGGACTTGATACGGTTACC	Gibson Assembly
Nev-LaM6 insert.R	cgggctttgttagcagccggtcgacttaGC TCACGGTTACCTGTGTACC	Gibson Assembly
Nev-LaMuniversal backbone.F	taaGTCGACCGGCTGCTAACAAA	Gibson Assembly
Nev-LaMuniversal backbone.R	taccagttgtacttgcgcACTGCTGCCACT CCCG	Gibson Assembly
UniversalLaM-Cwt backbone.F	GGTGGCAGTGGGAGTAGC	Gibson Assembly
UniversalLaM-Cwt backbone.R	ctagagggaaaccgttgtggGCTAGCCCAA AAAAACGGGTATG	Gibson Assembly
UniversalLaM-Cwt insert.F	atacccgtttttttgggctagcCCACAACG GTTTCCCTCTAGAAATA	Gibson Assembly
LaM3-Cwt insert.R	actcccgctacctccgctactcccactgcc accGTAACATTTGCTAACCGTCACTTGA	Gibson Assembly
LaM4-Cwt insert.R	actcccgctacctccgctactcccactgcc accAGTGAAAGGACTTGATACGGTTACC	Gibson Assembly
LaM6-Cwt insert.R	actcccgctacctccgctactcccactgcc accGCTCACGGTTACCTGTGTAC	Gibson Assembly
VHHUniversal-Cwt insert.F	ttgggctagcCCACAACGGTTTCCCTCTAG AAATAATTTT	Gibson Assembly
VHHE-Cwt insert.R	ctccgctactcccactgccaccTTCATGCC ACTCAATTTTCTGGG	Gibson Assembly
VHHE-Cwt backbone.F	cagaaaattgagtggcatgaaGGTGGCAGT GGGAGTAGCG	Gibson Assembly
VHHE-Cwt backbone.R	acaaaattatttctaGAGGGAAACCGTTGT GGGCTA	Gibson Assembly
VHHV-Cwt insert.R	cgctacctccgctactcccactgccaccGC TAGAGACTGTCACCTGTGTAC	Gibson Assembly
Nev-VHHE insert.F	gtagcgggagtggcagcagtCAGGTTCAGC TGGTGGAAAC	Gibson Assembly
Nev-VHHE insert.R	ttgttagcagccggtcgacttaTTCATGCC ACTCAATTTTCTGGG	Gibson Assembly
Nev-VHHE backbone.F	ccagaaaattgagtggcatgaaTAAGTCGA CCGGCTGCTAACAAAG	Gibson Assembly
Nev-VHHE backbone.R	ccggtttccaccagctgaacctgACTGCTG CCACTCCCGC	Gibson Assembly

VHHV-Nev insert.F	agtagcggaggtagcgggagtggcagcagtCAGGTCCAGTTAGTTGAAACAGGTG	Gibson Assembly
VHHV-Nev backbone.R	aactaactggacctgACTGCTGCCACTCCCGC	Gibson Assembly
Nev-FSR22 insert.F	gtagcgggagtggcagcagtGGGAGTTCTTCGAGCGGTAT	Gibson Assembly
Nev-FSR22 insert.R	tttgttagcagccggtcgacTTATGAGGAAGACTGCAAGATTTCCG	Gibson Assembly
Nev-FSR22 backbone.F	gaaatcttgcagtcttcctcaTAAGTCGACCGGCTGCTAACAAAG	Gibson Assembly
Nev-FSR22 backbone.R	cataccgctcgaagaactcccACTGCTGCCACTCCCGC	Gibson Assembly
NB03-Cwt insert.R	ctccgctactcccactgccaccCGACGAAACTGTTACCTGCG	Gibson Assembly
Linker28.F	AGCGGCGGATCTGGAAGCTCAggaggtagcgggagtgg	Blunt-ended ligation
Linker28.R	TGACCCGCTGCCTGAACCACCgctactcccactgccac	Blunt-ended ligation
Nev-Linker7.F	CAGGTTCAGTTAGTTGAATCCGGC	Blunt-ended ligation
Linker7-Cwt.F	AAAGCATTTATGCAAGTTGTCGAGGC	Blunt-ended ligation
Linker7.R	TCCGCTACTCCCACTGC	Blunt-ended ligation
Nb1-LacZ insert.F	tttgtttaactttaagaaggagatatacatATGCAGGTTCAGTTAGTTGAATCC	Gibson Assembly
Nb1-LacZ insert.R	gctaccgcctccaccagagcctcctccaccTTTACTAGAAACTGTTACTTGGGTGC	Gibson Assembly
UniversalLaG-LacZ backbone.F	GGTGGAGGAGGCTCTG	Gibson Assembly
UniversalLaG-LacZ backbone.R	CATATGTATATCTCCTTCTTAAAGTTAAACA	Gibson Assembly
LaG2-LacZ insert.F	tttgtttaactttaagaaggagatatacatATGGCGCAGGTGCAGC	Gibson Assembly
LaG2-LacZ insert.R	gctaccgcctccaccagagcctcctccaccTACTGTAACTTGTGTACCCTGGCCC	Gibson Assembly
LaG14-LacZ insert.F	ttaactttaagaaggagatatacatATGGCTCAAGTGCAGCTT	Gibson Assembly
LaG14-LacZ insert.R	cgcctccaccagagcctcctccaccGACGGTTACTTGTGTACCTTG	Gibson Assembly
Lag14-LacZ backbone.F	aaggtacacaagtaaccgtcGGTGGAGGAGGCTCTG	Gibson Assembly
LaG19-LacZ insert.F	tttgtttaactttaagaaggagatatacatATGGCGCAAGTGCAATTG	Gibson Assembly
LaG26-LacZ insert.F	tttgtttaactttaagaaggagatatacatATGGCACAGGTACAGCTTG	Gibson Assembly
LaG26-LacZ insert.R	gctaccgcctccaccagagcctcctccaccACTTACCGTGACTTGGGTG	Gibson Assembly

LaG27-LacZ insert.F	tttgtttaactttaagaaggagatatacat ATGGCAGATGTTCAGCTTGT	Gibson Assembly
UniversalLaM-LacZ insert.F	aaataattttgtttaactttaagaaggaga tatacatATGGCGCAAGTACAACTGGT	Gibson Assembly
LaM2-LacZ insert.R	gctaccgcctccaccagagcctcctccacc GATAGCCTCCTTTGATACCGTTACC	Gibson Assembly
LaM3-LacZ insert.R	gctaccgcctccaccagagcctcctccacc GTAACATTTGCTAACCGTCACTTGAG	Gibson Assembly
LaM4-LacZ insert.R	gctaccgcctccaccagagcctcctccacc AGTGAAAGGACTTGATACGGTTACC	Gibson Assembly
LaM6-LacZ insert.R	gctaccgcctccaccagagcctcctccacc GCTCACGGTTACCTGTGTACC	Gibson Assembly
LaM2-LacZ backbone.F	GGTGGAGGAGGCTCTGG	Gibson Assembly
LaM2-LacZ backbone.R	CATATGTATATCTCCTTCTTAAAGTTAAAC AAAATTATTTCT	Gibson Assembly

Table S7.

PCR primers used for plasmid cloning.

REFERENCES AND NOTES

1. K. N. Baker, M. H. Rendall, A. Patel, P. Boyd, M. Hoare, R. B. Freedman, D. C. James, Rapid monitoring of recombinant protein products: A comparison of current technologies. *Trends Biotechnol.* **20**, 149–156 (2002).

2. P. Yager, G. J. Domingo, J. Gerdes, Point-of-care diagnostics for global health. *Annu. Rev. Biomed. Eng.* **10**, 107–144 (2008).

3. The World Bank, World Bank Open Data, Current health expenditure per capita (current US$) (2023). https://data.worldbank.org/indicator/SH.XPD.CHEX.PC.CD.

4. C. Parolo, A. Sena-Torralba, J. F. Bergua, E. Calucho, C. Fuentes-Chust, L. Hu, L. Rivas, R. Álvarez-Diduk, E. P. Nguyen, S. Cinti, D. Quesada-González, A. Merkoçi, Tutorial: Design and fabrication of nanoparticle-based lateral-flow immunoassays. *Nat. Protoc.* **15**, 3788–3816 (2020).

5. D. Gasperino, T. Baughman, H. V. Hsieh, D. Bell, B. H. Weigl, Improving lateral flow assay performance using computational modeling. *Annu. Rev. Anal. Chem.* **11**, 219–244 (2018).

6. K. Pardee, A. A. Green, T. Ferrante, D. E. Cameron, A. DaleyKeyser, P. Yin, J. J. Collins, Paper-based synthetic gene networks. *Cell* **159**, 940–954 (2014).

7. P. L. Voyvodic, A. Pandi, M. Koch, I. Conejero, E. Valjent, P. Courtet, E. Renard, J.-L. Faulon, J. Bonnet, Plug-and-play metabolic transducers expand the chemical detection space of cell-free biosensors. *Nat. Commun.* **10**, 1697 (2019).

8. J. P. Hunt, E. L. Zhao, T. J. Free, M. Soltani, C. A. Warr, A. B. Benedict, M. K. Takahashi, J. S. Griffitts, W. G. Pitt, B. C. Bundy, Towards detection of SARS-CoV-2 RNA in human saliva: A paper-based cell-free toehold switch biosensor with a visual bioluminescent output. *N. Biotechnol.* **66**, 53–60 (2022).

9. M. P. McNerney, Y. Zhang, P. Steppe, A. D. Silverman, M. C. Jewett, M. P. Styczynski, Point-of-care biomarker quantification enabled by sample-specific calibration. *Sci. Adv.* **5**, eaax4473 (2019).

10. K. Y. Wen, L. Cameron, J. Chappell, K. Jensen, D. J. Bell, R. Kelwick, M. Kopniczky, J. C. Davies, A. Filloux, P. S. Freemont, A cell-free biosensor for detecting quorum sensing molecules in p. Aeruginosa-infected respiratory Samples. *ACS Synth. Biol.* **6**, 2293–2301 (2017).

11. W. Thavarajah, A. D. Silverman, M. S. Verosloff, N. Kelley-Loughnane, M. C. Jewett, J. B. Lucks, Point-of-use detection of environmental fluoride via a cell-free riboswitch-based biosensor. *ACS Synth. Biol.* **9**, 10–18 (2020).

12. K. Pardee, A. A. Green, M. K. Takahashi, D. Braff, G. Lambert, J. W. Lee, T. Ferrante, D. Ma, N. Donghia, M. Fan, N. M. Daringer, I. Bosch, D. M. Dudley, D. H. O'Connor, L. Gehrke, J. J. Collins, Rapid, low-cost detection of zika virus using programmable biomolecular components. *Cell* **165**, 1255–1266 (2016).

13. M. K. Takahashi, X. Tan, A. J. Dy, D. Braff, R. T. Akana, Y. Furuta, N. Donghia, A. Ananthakrishnan, J. J. Collins, A low-cost paper-based synthetic biology platform for analyzing gut microbiota and host biomarkers. *Nat. Commun.* **9**, 3347 (2018).

14. Y. Zhang, T. Kojima, G.-A. Kim, M. P. McNerney, S. Takayama, M. P. Styczynski, Protocell arrays for simultaneous detection of diverse analytes. *Nat. Commun.* **12**, 5724 (2021).

15. T. Pellinen, T. Huovinen, M. Karp, A cell-free biosensor for the detection of transcriptional inducers using firefly luciferase as a reporter. *Anal. Biochem.* **330**, 52–57 (2004).

16. J. K. Jung, K. K. Alam, M. S. Verosloff, D. A. Capdevila, M. Desmau, P. R. Clauer, J. W. Lee, P. Q. Nguyen, P. A. Pastén, S. J. Matiasek, J.-F. Gaillard, D. P. Giedroc, J. J. Collins, J. B. Lucks, Cell-free biosensors for rapid detection of water contaminants. *Nat. Biotechnol.* **38**, 1451–1459 (2020).

17. M. P. McNerney, F. Piorino, C. L. Michel, M. P. Styczynski, Active analyte import improves the dynamic range and sensitivity of a vitamin B_{12} biosensor. *ACS Synth. Biol.* **9**, 402–411 (2020).

18. F. Piorino, S. Johnson, M. P. Styczynski, A cell-free biosensor for assessment of hyperhomocysteinemia. *ACS Synth. Biol.* **12**, 2487–2492 (2023).

19. N. L. Anderson, The clinical plasma proteome: A survey of clinical assays for proteins in plasma and serum. *Clin. Chem.* **56**, 177–185 (2010).

20. M. Etzel, M. Mörl, Synthetic riboswitches: From plug and pray toward plug and play. *Biochemistry* **56**, 1181–1198 (2017).

21. H. Lee, T. Xie, B. Kang, X. Yu, S. W. Schaffter, R. Schulman, Plug-and-play protein biosensors using aptamer-regulated in vitro transcription. *Nat. Commun.* **15**, 7973 (2024).

22. G. E. Vezeau, L. R. Gadila, H. M. Salis, Automated design of protein-binding riboswitches for sensing human biomarkers in a cell-free expression system. *Nat. Commun.* **14**, 2416 (2023).

23. D.-M. Kim, J. R. Swartz, Efficient production of a bioactive, multiple disulfide-bonded protein using modified extracts of Escherichia coli. *Biotechnol. Bioeng.* **85**, 122–129 (2004).

24. L. A. Ryabova, D. Desplancq, A. S. Spirin, A. Plückthun, Functional antibody production using cell-free translation: Effects of protein disulfide isomerase and chaperones. *Nat. Biotechnol.* **15**, 79–84 (1997).

25. P. C. Fridy, Y. Li, S. Keegan, M. K. Thompson, I. Nudelman, J. F. Scheid, M. Oeffinger, M. C. Nussenzweig, D. Fenyö, B. T. Chait, M. P. Rout, A robust pipeline for rapid production of versatile nanobody repertoires. *Nat. Methods* **11**, 1253–1260 (2014).

26. K. Pardee, S. Slomovic, P. Q. Nguyen, J. W. Lee, N. Donghia, D. Burrill, T. Ferrante, F. R. McSorley, Y. Furuta, A. Vernet, M. Lewandowski, C. N. Boddy, N. S. Joshi, J. J. Collins, Portable, on-demand biomolecular manufacturing. *Cell* **167**, 248–259.e12 (2016).

27. E. Y. Yang, K. Shah, Nanobodies: Next generation of cancer diagnostics and therapeutics. *Front. Oncol.* **10**, 1182 (2020).

28. C. McMahon, A. S. Baier, R. Pascolutti, M. Wegrecki, S. Zheng, J. X. Ong, S. C. Erlandson, D. Hilger, S. G. F. Rasmussen, A. M. Ring, A. Manglik, A. C. Kruse, Yeast surface display platform for rapid discovery of conformationally selective nanobodies. *Nat. Struct. Mol. Biol.* **25**, 289–296 (2018).

29. X. Chen, M. Gentili, N. Hacohen, A. Regev, A cell-free nanobody engineering platform rapidly generates SARS-CoV-2 neutralizing nanobodies. *Nat. Commun.* **12**, 5506 (2021).

30. L. Gieselmann, C. Kreer, M. S. Ercanoglu, N. Lehnen, M. Zehner, P. Schommers, J. Potthoff, H. Gruell, F. Klein, Effective high-throughput isolation of fully human antibodies targeting infectious pathogens. *Nat. Protoc.* **16**, 3639–3671 (2021).

31. J. Pu, J. Zinkus-Boltz, B. C. Dickinson, Evolution of a split RNA polymerase as a versatile biosensor platform. *Nat. Chem. Biol.* **13**, 432–438 (2017).

32. J. Pu, K. Kentala, B. C. Dickinson, Multidimensional control of Cas9 by evolved RNA polymerase-based biosensors. *ACS Chem. Biol.* **13**, 431–437 (2018).

33. M. H. Kubala, O. Kovtun, K. Alexandrov, B. M. Collins, Structural and thermodynamic analysis of the GFP:GFP-nanobody complex. *Protein Sci.* **19**, 2389–2401 (2010).

34. A. Koide, J. Wojcik, R. N. Gilbreth, R. J. Hoey, S. Koide, Teaching an old scaffold new tricks: Monobodies constructed using alternative surfaces of the FN3 scaffold. *J. Mol. Biol.* **415**, 393–405 (2012).

35. M. Brauchle, S. Hansen, E. Caussinus, A. Lenard, A. Ochoa-Espinosa, O. Scholz, S. G. Sprecher, A. Plückthun, M. Affolter, Protein interference applications in cellular and developmental biology using DARPins that recognize GFP and mCherry. *Biol. Open* **3**, 1252–1261 (2014).

36. J. Pu, M. Disare, B. C. Dickinson, Evolution of C-terminal modification tolerance in full-length and split T7 RNA polymerase biosensors. *Chembiochem* **20**, 1547–1553 (2019).

37. A. T. Q. Cong, T. L. Witter, M. J. Schellenberg, High-efficiency recombinant protein purification using mCherry and YFP nanobody affinity matrices. *Protein Sci.* **31**, e4383 (2022).

38. J. Garamella, R. Marshall, M. Rustad, V. Noireaux, The all *E. coli* TX-TL toolbox 2.0: A platform for cell-free synthetic biology. *ACS Synth. Biol.* **5**, 344–355 (2016).

39. D. Garenne, S. Thompson, A. Brisson, A. Khakimzhan, V. Noireaux, The all-E. coliTXTL toolbox 3.0: New capabilities of a cell-free synthetic biology platform. *Synth. Biol.* **6**, ysab017 (2021).

40. P.-A. Koenig, H. Das, H. Liu, B. M. Kümmerer, F. N. Gohr, L.-M. Jenster, L. D. J. Schiffelers, Y. M. Tesfamariam, M. Uchima, J. D. Wuerth, K. Gatterdam, N. Ruetalo, M. H. Christensen, C. I. Fandrey, S. Normann, J. M. P. Tödtmann, S. Pritzl, L. Hanke, J. Boos, M. Yuan, X. Zhu, J. L. Schmid-Burgk, H. Kato, M. Schindler, I. A. Wilson, M. Geyer, K. U. Ludwig, B. M. Hällberg, N. C. Wu, F. I. Schmidt, Structure-guided multivalent nanobodies block SARS-CoV-2 infection and suppress mutational escape. *Science* **371**, eabe6230 (2021).

41. V. Chonira, Y. D. Kwon, J. Gorman, J. B. Case, Z. Ku, R. Simeon, R. G. Casner, D. R. Harris, A. S. Olia, T. Stephens, L. Shapiro, M. F. Bender, H. Boyd, I.-T. Teng, Y. Tsybovsky, F. Krammer, N. Zhang, M. S. Diamond, P. D. Kwong, Z. An, Z. Chen, A potent and broad neutralization of SARS-CoV-2 variants of concern by DARPins. *Nat. Chem. Biol.* **19**, 284–291 (2023).

42. S. Stanley, D. J. Hamel, I. D. Wolf, S. Riedel, S. Dutta, E. Contreras, C. J. Callahan, A. Cheng, R. Arnaout, J. E. Kirby, P. J. Kanki, Limit of detection for rapid antigen testing of the SARS-CoV-2 omicron and delta variants of concern using live-virus culture. *J. Clin. Microbiol.* **60**, e00140-22 (2022).

43. S. Bharadwaj, S. Ginoya, P. Tandon, T. D. Gohel, J. Guirguis, H. Vallabh, A. Jevenn, I. Hanouneh, Malnutrition: Laboratory markers vs nutritional assessment. *Gastroenterol. Rep.* **4**, 272–280 (2016).

44. O. Müller, M. Krawinkel, Malnutrition and health in developing countries. *CMAJ* **173**, 279–286 (2005).

45. W. Drammeh, N. A. Hamid, A. J. Rohana, Determinants of household food insecurity and its association with child malnutrition in sub-saharan Africa: A review of the literature. *Curr. Res. Nutrition Food Sci. J.* **7**, 610–623 (2019).

46. D. J. Corsi, M. Neuman, J. E. Finlay, S. Subramanian, Demographic and health surveys: A profile. *Int. J. Epidemiol.* **41**, 1602–1613 (2012).

47. L. Ma, Y. Sun, X. Kang, Y. Wan, Development of nanobody-based flow injection chemiluminescence immunoassay for sensitive detection of human prealbumin. *Biosens. Bioelectron.* **61**, 165–171 (2014).

48. C. J. Hood, N. S. Hendren, R. Pedretti, L. R. Roth, L. Saelices, J. L. Grodin, Update on disease-specific biomarkers in transthyretin cardiac amyloidosis. *Curr. Heart Fail. Rep.* **19**, 356–363 (2022).

49. A. W. Brookwell, J. L. Gonzalez, A. W. Martinez, J. P. Oza, Development of solid-state storage for cell-free expression systems. *ACS Synth. Biol.* **12**, 2561–2577 (2023).

50. K. M. Wilding, E. L. Zhao, C. C. Earl, B. C. Bundy, Thermostable lyoprotectant-enhanced cell-free protein synthesis for on-demand endotoxin-free therapeutic production. *N. Biotechnol.* **53**, 73–80 (2019).

51. K. F. Warfel, A. Williams, D. A. Wong, S. E. Sobol, P. Desai, J. Li, Y.-F. Chang, M. P. DeLisa, A. S. Karim, M. C. Jewett, A low-cost, thermostable, cell-free protein synthesis platform for on-demand production of conjugate vaccines. *ACS Synth. Biol.* **12**, 95–107 (2023).

52. A. Huang, P. Q. Nguyen, J. C. Stark, M. K. Takahashi, N. Donghia, T. Ferrante, A. J. Dy, K. J. Hsu, R. S. Dubner, K. Pardee, M. C. Jewett, J. J. Collins, BioBits™ Explorer: A modular synthetic biology education kit. *Sci. Adv.* **4**, eaat5105 (2018).

53. S. M. Blum, M. S. Lee, G. E. Mgboji, V. L. Funk, K. Beabout, S. V. Harbaugh, P. A. Roth, A. T. Liem, A. E. Miklos, P. A. Emanuel, S. A. Walper, J. L. Chávez, M. W. Lux, Impact of porous matrices and concentration by lyophilization on cell-free expression. *ACS Synth. Biol.* **10**, 1116–1131 (2021).

54. S. M. Namaste, G. J. Aaron, R. Varadhan, J. M. Peerson, P. S. Suchdev, Methodologic approach for the biomarkers reflecting inflammation and nutritional determinants of anemia (BRINDA) project. *Am. J. Clin. Nutr.* **106**, 333S–347S (2017).

55. T. H. Segall-Shapiro, A. J. Meyer, A. D. Ellington, E. D. Sontag, C. A. Voigt, A 'resource allocator' for transcription based on a highly fragmented T7 RNA polymerase. *Mol. Syst. Biol.* **10**, 742 (2014).

56. D. G. Gibson, L. Young, R.-Y. Chuang, J. C. Venter, C. A. Hutchison, H. O. Smith, Enzymatic assembly of DNA molecules up to several hundred kilobases. *Nat. Methods* **6**, 343–345 (2009).

57. K. A. Datsenko, B. L. Wanner, One-step inactivation of chromosomal genes in Escherichia coli K-12 using PCR products. *Proc. Natl. Acad. Sci. U.S.A.* **97**, 6640–6645 (2000).

58. Y.-C. Kwon, M. C. Jewett, High-throughput preparation methods of crude extract for robust cell-free protein synthesis. *Sci. Rep.* **5**, 8663 (2015).

59. K. Hajian-Tilaki, Receiver operating characteristic (ROC) curve analysis for medical diagnostic test evaluation. *Caspian J. Intern. Med.* **4**, 627–635 (2013).

60. L. A. Gross, G. S. Baird, R. C. Hoffman, K. K. Baldridge, R. Y. Tsien, The structure of the chromophore within DsRed, a red fluorescent protein from coral. *Proc. Natl. Acad. Sci. U.S.A.* **97**, 11990–11995 (2000).

61. C. Klumpp-Thomas, H. Kalish, M. Drew, S. Hunsberger, K. Snead, M. P. Fay, J. Mehalko, A. Shunmugavel, V. Wall, P. Frank, J.-P. Denson, M. Hong, G. Gulten, S. Messing, J. Hicks, S. Michael, W. Gillette, M. D. Hall, M. J. Memoli, D. Esposito, K. Sadtler, Standardization of ELISA protocols for serosurveys of the SARS-CoV-2 pandemic using clinical and at-home blood sampling. *Nat. Commun.* **12**, 113 (2021).

62. T. S. M. de Assis, M. L. Freire, J. de Pina Carvalho, A. Rabello, G. Cota, Cost-effectiveness of anti-SARS-CoV-2 antibody diagnostic tests in Brazil. *PLOS ONE* **17**, e0264159 (2022).

63. A. Biby, X. Wang, X. Liu, O. Roberson, A. Henry, X. Xia, Rapid testing for coronavirus disease 2019 (COVID-19). *MRS Commun.* **12**, 12–23 (2022).

64. Z. Z. Sun, C. A. Hayes, J. Shin, F. Caschera, R. M. Murray, V. Noireaux, Protocols for implementing an *Escherichia coli* based TX-TL cell-free expression system for synthetic biology. *J. Vis. Exp.* e50762 (2013).

65. C.-L. Hsieh, J. A. Goldsmith, J. M. Schaub, A. M. DiVenere, H.-C. Kuo, K. Javanmardi, K. C. Le, D. Wrapp, A. G. Lee, Y. Liu, C.-W. Chou, P. O. Byrne, C. K. Hjorth, N. V. Johnson, J. Ludes-Meyers, A. W. Nguyen, J. Park, N. Wang, D. Amengor, J. J. Lavinder, G. C. Ippolito, J. A. Maynard, I. J. Finkelstein, J. S. McLellan, Structure-based design of prefusion-stabilized SARS-CoV-2 spikes. *Science* **369**, 1501–1505 (2020).

66. J. Pallesen, N. Wang, K. S. Corbett, D. Wrapp, R. N. Kirchdoerfer, H. L. Turner, C. A. Cottrell, M. M. Becker, L. Wang, W. Shi, W.-P. Kong, E. L. Andres, A. N. Kettenbach, M. R. Denison, J. D. Chappell, B. S. Graham, A. B. Ward, J. S. McLellan, Immunogenicity and structures of a rationally designed prefusion MERS-CoV spike antigen. *Proc. Natl. Acad. Sci. U.S.A.* **114**, E7348–E7357 (2017).
